# DRR Dhan 58, a Seedling Stage Salinity Tolerant NIL of Improved Samba Mahsuri Shows Superior Performance in Multi-location Trials

**DOI:** 10.1186/s12284-022-00591-3

**Published:** 2022-08-17

**Authors:** G. Rekha, V. Abhilash Kumar, C. G. Gokulan, M. B. V. N. Koushik, B. Laxmi Prasanna, Swapnil Kulkarni, D. Aleena, G. Harika, S. K. Hajira, K. Pranathi, E. Punniakoti, R. R. Kale, T. Dilip Kumar, D. Ayyappa, M. Anila, Pragya Sinha, K. K. Manohara, G. Padmavathi, L. V. Subba Rao, G. S. Laha, M. S. Srinivas Prasad, R. A. Fiyaz, K. Suneetha, S. M. Balachandran, Hitendra Kumar Patel, Ramesh V. Sonti, P. Senguttuvel, R. M. Sundaram

**Affiliations:** 1 Department of Biotechnology, ICAR-Indian Institute of Rice Research, Rajendranagar, Hyderabad, India; 2 Rallis India Limited, Seeds/Biotech R&D Division, Bangalore, India; 3 Department of Plant Breeding, RARS, PJTSAU, Jagtial, India; 4 Genetics and Plant Breeding, ICAR- Central Coastal Agricultural Research Institute, Ella, Goa India; 5 Crop Improvement Section, CSIR-Centre for Cellular and Molecular Biology, Hyderabad, India; 6 Department of Biology, Indian Institute of Science Education and Research, Tirupati, India

**Keywords:** Rice, Salinity tolerance, Bacterial blight, Marker-assisted selection, Whole genome resequencing, *Saltol*

## Abstract

**Background:**

Improved Samba Mahsuri (ISM) is an elite, high-yielding, bacterial blight resistant, fine-grained rice variety with low glycaemic index. It is highly sensitive to salt stress, particularly at seedling stage, which significantly reduces its yield potential in coastal areas. A salinity tolerant QTL*, Saltol,* associated with seedling stage tolerance was previously mapped on chromosome 1 (10.6–11.5 Mb) from the Indian landrace, Pokkali and is effective in different genetic backgrounds. The objective of this study was to enhance salinity tolerance of ISM by incorporating the *Saltol* QTL through marker-assisted backcross breeding using the breeding line, FL478 (Pokkali/IR29).

**Results:**

Foreground selection was carried out at each generation using five *Saltol-*specific markers and three bacterial blight resistance genes, *Xa21, xa13* and *xa5*. Background selection was conducted using 66 well distributed polymorphic SSR markers and at the BC_3_F_2_ generation, a single plant with maximum recurrent parent genome recovery (95.3%) was identified and advanced to the BC_3_F_4_ generation. Based on bacterial blight resistance, seedling stage salinity tolerance and resemblance to ISM, four advanced breeding lines were selected for testing in replicated experiments near Hyderabad, India. A promising near-isogenic line, DRR Dhan 58, was evaluated in multi-location trials-coastal salinity and it showed significant salinity tolerance, resistance to bacterial blight disease, high yield and excellent grain quality during the 2019 and 2020 trials. DRR Dhan 58 was 95.1% similar to ISM based on genotyping with the 90 K SNP chip. Whole genome resequencing analysis of Pokkali and FL478 which were salinity tolerant checks, ISM and DRR Dhan 58 showed a high degree of relatedness with respect to the candidate gene loci for *Saltol* and *OsSKC1* (*Shoot K*^+^ *Concentration 1*).

**Conclusion:**

DRR Dhan 58, possessing *Saltol* and three bacterial blight resistance genes (*Xa21, xa13* and *xa5*) in the genetic background of the Indian mega-variety of rice, Samba Mahsuri, was developed for potential cultivation in areas prone to seedling stage salinity, as well as areas with endemic bacterial blight disease. This entry had a 24% yield advantage over the recurrent parent ISM under coastal saline conditions in multi-location trials and was recently released for commercial cultivation in India.

**Supplementary Information:**

The online version contains supplementary material available at 10.1186/s12284-022-00591-3.

## Introduction

A rapidly changing climate and increasing incidence of major biotic and abiotic stresses pose an imminent threat to global rice production and food security (Ahmad et al. 2019), necessitating the development of rice varieties, which possess durable resistance or tolerance to various stresses (Hasan et al. 2015). One way to achieve this would be by stacking genes/QTLs that confer broad spectrum and durable resistance or tolerance in the background of high yielding varieties through marker-assisted breeding. Using this strategy, rice varieties that survive multiple pathogens or pest races, as well as exhibiting yield superiority even under unfavorable environmental conditions can be developed (Sandhu et al. 2019).

Among the biotic stresses, bacterial blight, caused by *Xanthomonas oryzae* pv. *oryzae* (*Xoo*), is one of the most widespread diseases that causes significant damage and leads to drastic yield losses (Babujee and Gnanamanickham 2000; Pradhan et al. 2015). To date, at least 46 genes conferring resistance against bacterial blight have been identified (Zheng et al. 2016; Neelam et al. 2020). The genes vary in their resistance levels due to significant variability among *Xoo* races and the emergence of virulent strains. Hence, pyramiding of two or more genes conferring resistance against this disease is the best strategy to develop durable resistance in rice (Das et al. 2015; Pradhan et al. 2015).

Among the abiotic stresses, salinity is one of the most important constraints affecting significant area under rice cultivation in many rice growing countries including India. Salinity stress causes severe damage during the seedling and reproductive stages of crop growth, causing yield losses up to 50% (Molla et al. 2015), while the vegetative stage remains unaffected (Zeng and Shannon 2000). Tolerance to salinity is a complex trait, involving several genes as well as different biochemical and physiological mechanisms, such as sodium exclusion from roots, controlled sodium transport between root and shoot, and sequestering sodium in older tissues and vacuoles (Hoang et al. 2016). A few quantitative trait loci (QTLs) and genes governing salt tolerance were reported in rice of which *Saltol,* a major QTL conferring seedling stage salinity tolerance was the most significant (Thomson et al. 2010). *Saltol* was discovered in Pokkali, a salinity tolerant Indian landrace and introgressed into FL478, located between 10.6 and 11.5 Mb on chromosome 1 (Thomson et al. 2010; Kim et al. 2009; Ashutosh et al. 2020) and imparts salt tolerance by regulating Na^+^/K^+^ homeostasis under salt stress (Thomson et al. 2010). Ren et al. (2005) demonstrated the *Shoot K*^+^ *Concentration-1* (*SKC1*) gene, *OsSKC1*, which encodes a sodium transporter, is the source determinant of salt tolerance in the *Saltol* region.

Genomics-based technologies are increasingly used for accelerating rice breeding programs, increasing their precision and speed (Zhou et al. 2013). Several breeder ready chips are available in various crops like rice for use in indirect selection of target genes/QTLs and also background profiling (Yu et al. 2014; Chen et al. 2014). Furthermore, the availability of a high-quality reference genome of rice along with the genomes of > 3000 rice accessions allows quick and precise alignment, mapping and annotation of sequences emanating from next generation sequencing studies and identification of variants (Nguyen et al. 2018; Huang et al. 2013; McNally et al. 2009; Yamamoto et al. 2010; Arai-Kichise et al. 2011).

The rice variety Improved Samba Mahsuri (ISM) is a near-isogenic line (NIL) of the highly popular Indian mega-rice variety, Samba Mahsuri with a high level of bacterial blight disease resistance because the three major resistance genes, *Xa21, xa13* and *xa5* are introgressed (Sundaram et al. 2008). ISM is classified as a fine-grain type and has high-yield and excellent cooking and eating qualities like its recurrent parent, Samba Mahsuri. A recent study carried out by ICMR-National Institute of Nutrition, Hyderabad, India concluded that ISM has a very low glycaemic index (50.99), ensuring slow release of glucose into the blood stream and hence, it is highly suitable for people with Type II diabetes. This has enhanced its market potential and profitability (Sundaram et al. 2018). Due to these properties, ISM is steadily replacing Samba Mahsuri, particularly in areas endemic to bacterial blight in South India and is currently estimated to be cultivated on approximately 300,000 ha. One of the major limitations of ISM is its high susceptibility to seedling stage salinity stress, which limits its spread in the coastal areas of India.

The objective of this study was to enhance the seedling stage salinity tolerance of ISM through marker-assisted backcross breeding for the development of NILs possessing *Saltol*. Subsequently, we attempted to validate a promising NIL of ISM, DRR Dhan 58 for salinity tolerance and bacterial blight resistance through multi-location trials.

## Results

### Marker-Assisted introgression of *Saltol* QTL into Improved Samba Mahsuri

**Fig. 1:**
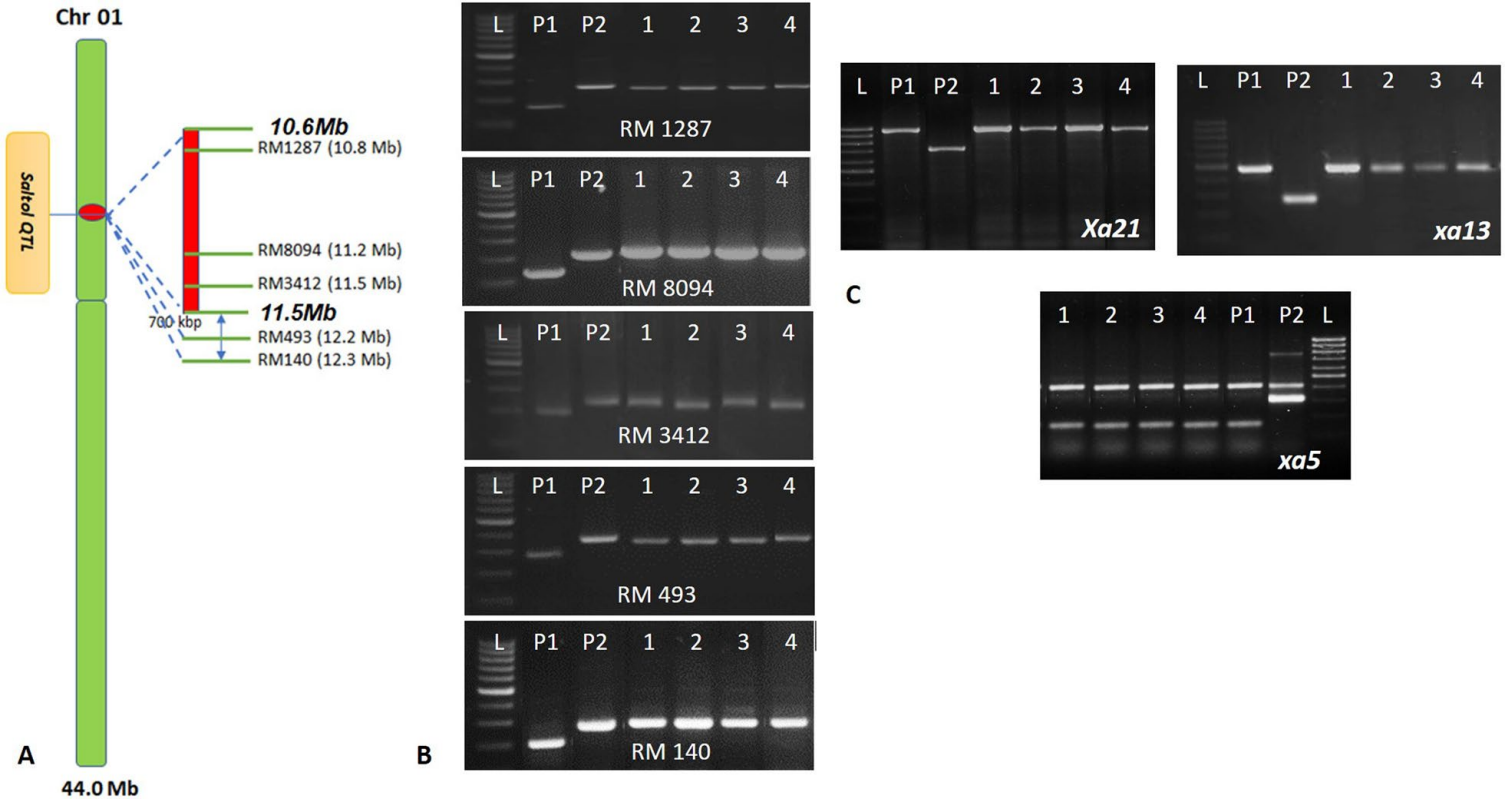
**A**: Representation of *Saltol* QTL region and the markers spanning along the target region **B**- Confirmation of promising BC_3_F_4_ lines with the five markers spanning the *Saltol* QTL. L- 100bpLadder; P1- ISM, P2- FL478; Samples 1–4–Introgression lines of ISM. C- Confirmation of promising BC_3_F_4_ lines for BB resistance genes (*Xa21, xa13* and *xa5*). L- 100bpLadder; P1-ISM, P2- FL478; Samples 1–4–Introgression lines of ISM. 1- RP6287-43; 2-RP6287-88; RP6287-12; RP6287-178

From the cross, ISM x FL478, 41 true F_1_ plants were identified though foreground selection and subsequent backcrossing to ISM produced 312 BC_1_F_1_ plants (Additional file 1: Fig. S1). Foreground selection revealed 120 plants were heterozygous for all five *Saltol* specific markers and 16 of these were homozygous for the three bacterial blight resistance genes, *Xa21*, *xa13* and *xa5*. These 16 plants were screened with 66 genome-wide polymorphic SSR markers (Additional file 2: Table S1), out of which 51 were homozygous for recurrent parent allele; one plant, No. RP6287-188, with highest ISM parent genome recovery (76.2%) was backcrossed to generate 123 BC_2_F_1_ plants. Foreground selection revealed 56 plants with the *Saltol* QTL and background selection of best 15 plants selected, it was found that 58 markers were homozygous for recurrent parent allele for BC_2_F_1_ plant No. RP6287-188–45 with 87.5% ISM genome recovery, which was then backcrossed to produce 183 BC_3_F_1_ plants. Among them, 86 carried *Saltol* and background selection of 15 plants selected based on morphological similarity; it was observed that 60 markers were showing homozygous recurrent parent allele for plant No. RP6287-188-45-12 with 91.0% ISM genome recovery which was selfed to produce 532 BC_3_F_2_ progeny. Genotyping with five *Saltol* SSR markers identified 280 plants homozygous for the *Saltol* (Fig. 1) and background selection was carried out with best 10 plants selected; 63 markers showed homozygous positive allele for plant No. RP6287-188-45-12-88 with 95.3% ISM genome recovery. This plant, had 1.0 Mb segment at the proximal end and a 1.5 Mb segment at the distal end of the *Saltol* region which were introgressed from the donor parent, FL478. Thus, based on analysis with genome-wide SSR markers, a 2.5 Mb segment from FL478 (Additional file 1: Figure S2) was introgressed in the selected BC_3_F_2_ plant along with *Saltol*. This plant was selfed and four lines (RP6287-12, RP6287-43, RP6287-88 and RP6287-178) were selected from the BC_3_F_4_ progeny based on similarity in morphological traits and grain type with respect to ISM under field conditions.

### Assessment of Seedling Stage Salinity Tolerance

**Fig. 2:**
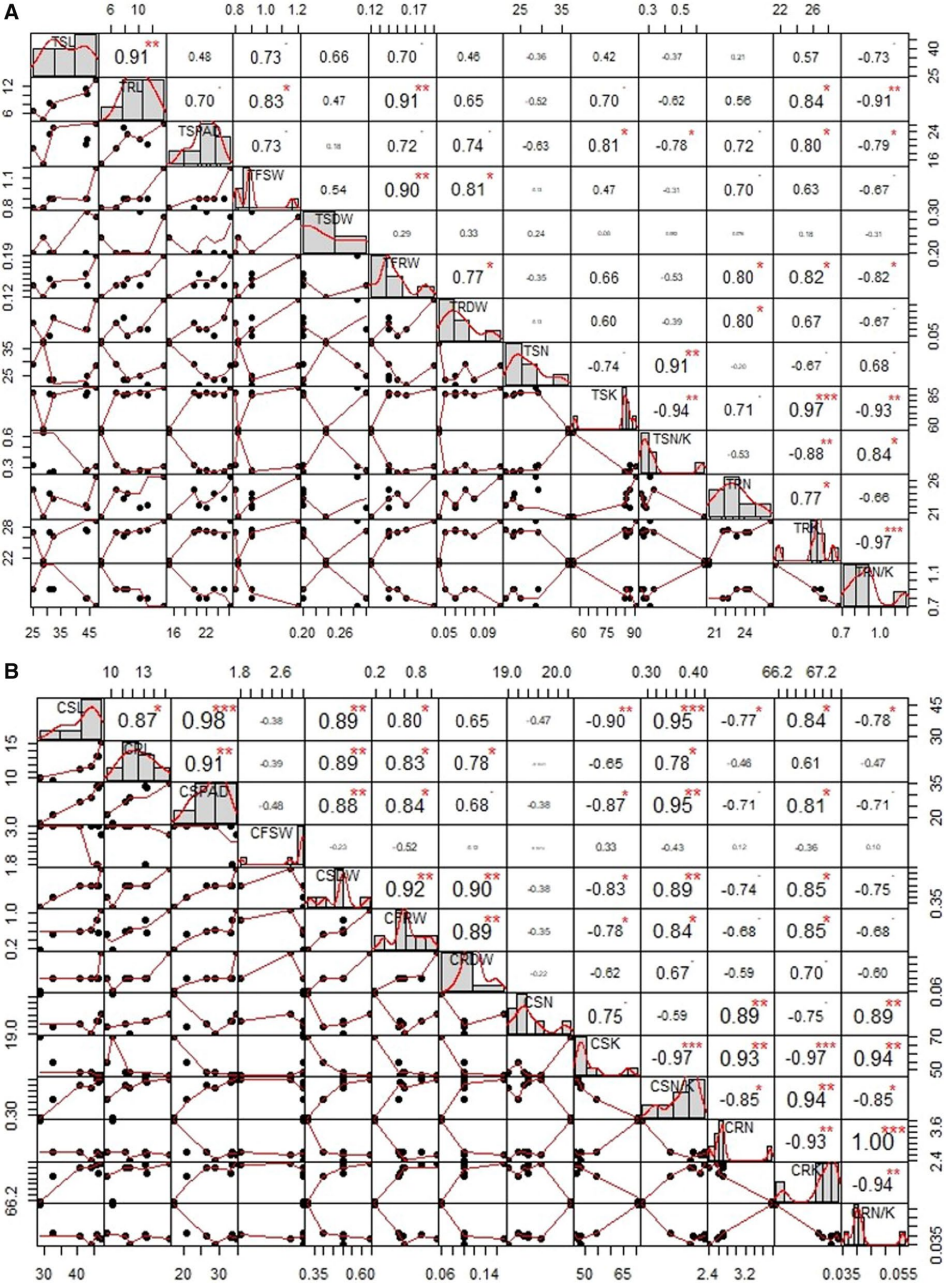
**A**: Correlation plot for seedling stage salinity screening under stressed conditions. TSL-Treated shoot length; TSPAD-Treated SPAD; TSFW- Treated fresh shoot weight; TSDW- Treated shoot dry weight; TSN-Treated shoot sodium; TSK- Treated shoot potassium; TSN/K- Treated shoot Na + /K + ratio; TRL-Treated root length; TRFW- Treated fresh root weight; TRDW- Treated root dry weight; TRN-Treated root sodium; TSK- Treated root potassium; TRN/K- Control root Na + /K + ratio. The diagonal represents the distribution of each variable. The top of the diagonal indicates the correlation values and also significance levels as stars (Each significance level is associated to a p-value—0.0001***, 0.01**, 0.05*, 0.1., 1) while the bottom diagonal represents the bivariate scatter plots with a fitted line. **B**: Correlation plot for seedling stage salinity screening under unstressed conditions. CSL-Control shoot length; CSPAD-Control SPAD; CSFW- Control fresh shoot weight; CSDW- Control shoot dry weight; CSN-Control shoot sodium; CSK- Control shoot potassium; CSN/K- Control shoot Na + /K + ratio; CRL-Control root length; CRFW- Control fresh root weight; CRDW- Control root dry weight; CRN-Control root sodium; CSK- Control root potassium; CRN/K- Control root Na + /K + ratio. The diagonal represents the distribution of each variable. The top of the diagonal indicates the correlation values and also significance levels as stars (Each significance level is associated to a p-value—0.0001***, 0.01**, 0.05*, 0.1., 1) while the bottom diagonal represents the bivariate scatter plots with a fitted line.

The four selected introgressed lines of ISM showed high level of seedling stage salinity tolerance with a score of 1 to 3, which was comparable to that of FL478 under a salt stress of 12 deci Siemens per metre (dSm^−1^) (i. e. 120 mM of NaCl) for two weeks (Additional file 1: Figure S3). In contrast, the recurrent parent, ISM was highly sensitive with a score of 9. Under stressed conditions ISM had significantly higher Na^+^ concentration in both shoot and root as compared to FL478; while K^+^ content was lower in ISM (Additional file 3: Table S2A) while high in FL478. All the selected backcross derived lines of ISM showed higher K^+^ levels with mean values ranging from 85.0 to 87.4 mg/g in shoots; 67.2 to 67.5 mg/g in roots and lower Na^+^ levels with mean values ranging from 19.0 to 19.4 mg/g in shoots; 12.4 to 12.7 mg/g in roots, which was similar to the tolerant parent, i.e., FL478 (Fig. 2A; Additional file 3: Table S2A). Under unstressed conditions, the root and shoot Na^+^ and K^+^ content of all the tested lines were identical (Additional file 3: Table S2B). Under stressed conditions, a strong positive correlation was observed between shoot length and root K^+^ content (0.84) as well with shoot Na^+^/K^+^ ratio (0.95); between root length and root K^+^ content (0.61) as well with shoot Na^+^/K^+^ ratio (0.78). However, no significant association was observed between the ionic contents of shoot and root with other parameters under unstressed conditions (Fig. 2B).

### Assessment of Improved Lines for Bacterial Blight Resistance

The recurrent parent, ISM possessing *Xa21, xa13* and *xa5* exhibited high level of bacterial blight disease resistance with a lesion length < 3 cm (SES score 1) while FL478, the donor parent was susceptible with a 9 cm lesion length (SES score 9). All four introgressed lines were resistant to bacterial blight, exhibiting resistance similar to the recurrent parent with a lesion length < 3 cm (SES score 1) (Table 1).

**Table 1:** Phenotypic screening for bacterial blight (BB) resistance in improved lines of ISM

S. No	Plant identity	Allelic status For the target traits	Average lesion length for BB^#^	*SES score
1	ISM	*Xa21Xa21,xa13xa13,xa5xa5*	0.0 ± 0.0	1
2	TN1 (Susceptible check for BB)	–	8.7 ± 0.3	9
3	RP6287-12	*Xa21Xa21, xa13xa13, xa5xa5, Saltol*	1.7 ± 0.7	3
4	RP6287-43	*Xa21Xa21, xa13xa13, xa5xa5, Saltol*	1.3 ± 0.9	3
5	RP6287-88	*Xa21Xa21, xa13xa13, xa5xa5, Saltol*	0.3 ± 0.3	3
6	RP6287-178	*Xa21Xa21, xa13xa13, xa5xa5, Saltol*	2.3 ± 0.7	3

*SES—Standard Evaluation System;

^#^All the introgression lines exhibited bacterial blight resistance with DRR isolate DX0-20

### Evaluation of Backcross Derived Lines of ISM for Agro-Morphological Traits

The improved lines at BC_3_F_5_ generation possessing *Saltol* had equivalent or higher grain yield (21.1 to 22.5 g per plant) as compared to ISM (20.2 g), equivalent or higher thousand grain weight (19.1 to 19.7 g) as compared to ISM (18.0 g), taller (84.7 to 86.7 cm) as compared to ISM (82.7 cm) and with more number of productive tillers (15 to 17 no.) as compared to ISM (13 no.) (Additional file 1: Fig. S3A; Table 2). Two lines No. RP6287-43 and No. R6287-12, were equivalent to that of ISM (20.0 cm) in terms of panicle length, while the other two improved lines were observed to have longer panicles (23.8 and 24.0 cm) as compared to the recurrent parent. Days to 50% flowering (DFF) of all the improved lines were similar to ISM, except RP6287-88 (96 days) which flowered a week earlier than the recurrent parent (102 days). All the improved lines had fully exserted panicles, while the recurrent parent show partial exsertion of the panicles.

**Table 2:** Analysis of agro-morphological characters of ISM introgressed lines for salinity tolerance along with parents under field conditions at ICAR-IIRR (Wet season, June to November 2018)

S.No	Plant identity^#^	Days to 50% flowering (DFF)	Mean plant height (cm)	No. of productive panicles /plant	Panicle length (cm)	1000 seed weight (gm)	Grain yield per plant (gm)	Panicle exsertion
1	ISM	102 ± 2.0	82.7 ± 1.8	13.0 ± 0.6	20.0 ± 1.0	18.0 ± 1.2	20.2 ± 1.1	PE
2	FL478	124 ± 2.0	101.0 ± 2.9	8.0 ± 0.6	16.0 ± 1.0	16.3 ± 0.9	19.2 ± 0.9	FE
3	RP6287-12	101.7 ± 1.2	86.3 ± 3.5	17.0 ± 0.6	20.3 ± 1.5	19.1 ± 1.2	21.6 ± 0.6	FE
4	RP6287-43	106.3 ± 1.5	85.7 ± 2.3	15.0 ± 0.6	20.1 ± 2.0	19.3 ± 0.9	21.1 ± 0.9	FE
5	RP6287-88	96.3 ± 2.1	84.7 ± 3.2	17.0 ± 0.6	24.0 ± 3.1	19.3 ± 1.2	22.5 ± 0.7	FE
6	RP6287-178	101.3 ± 1.5	86.7 ± 2.3	17.0 ± 0.6	23.8 ± 1.0	19.7 ± 1.0	21.5 ± 0.8	FE
	**CD%**	**14.42**	**12.12**	**3.68**	**3.17**	**3.27**	**2.52**	
	**CV%**	**8.03**	**7.98**	**6.58**	**8.81**	**10.14**	**6.36**	
	**PCV**	**14.98**	**12.16**	**23.03**	**8.21**	**8.10**	**8.95**	
	**GCV**	**12.51**	**9.66**	**22.07**	**6.15**	**6.57**	**7.94**	
	***F***	**51.42**	**7.46**	**34.72**	**4.48**	**3.47**	**42.44**	
	***P value* (< 0.05)**	** < 0.0001**	**0.00168**	** < 0.0001**	** < 0.0113**	** < 0.0318**	** < 0.0001**	

^#^ISM-Recurrent parent; FL478-Donor parent; CV-Coefficient of variance; CD-Critical differential at 5%; PCV-Phenotypic coefficient of variation; GCV-Genotypic coefficient of variation; p value-probability level; ± -Standard error and values given are mean of three replications; PE- Partial exsertion; FE- Fully exserted

All the four selected improved lines showed better or on par yield with ISM; among them RP6287-88 performed well in terms of yield attributes in comparison to ISM and other introgression lines

### Assessment of Grain and Cooking Quality Attributes

Evaluation of the four ISM NILs and parents for grain size [kernel length, kernel width, length to width ratio, grain chalkiness and volume expansion ratio (VER)] and cooking quality parameters [kernel length after cooking (KLAC), elongation ratio (ER), alkali spreading Value (ASV), amylose content, gel consistency (mm)], showed that all the NILs were similar to ISM (Table 3). All the NILs, except RP6287-178 possessed medium-slender grain type similar to the recurrent parent. Furthermore, kernel length after cooking and elongation ratio of the improved versions were also observed to be similar to that of ISM with low gelatinization temperature as indicated by the alkali spreading value of 4.0.

**Table 3:** Analysis of grain size and cooking quality characters of the parents and four improved lines of ISM

Samples	KL*	KW*	L/W*	Grain chalkiness	VER*	Water content	KLAC*	ER*	ASV*	AC*	GC*	Grain type
FL478	6.31	2.14	2.94	OC	4.6	210	12.4	1.96	4	26.54	22	MS
ISM	4.45	1.75	2.54	VOC	4.1	195	7.7	1.73	4	23.34	22	MS
RP6287-12	4.76	1.89	2.62	VOC	4.7	205	8.5	1.8	4	26.98	38	MS
RP6287-43	5.45	1.89	2.88	VOC	5.0	155	8.2	1.5	4	24.75	22	MS
RP6287-88	4.72	1.81	2.45	VOC	4.6	200	7.8	1.63	4	24.69	22	MS
RP6287-178	5.65	1.92	2.98	OC	4.7	200	10.6	1.87	4	24.90	23	MB

*KL- Kernel length (mm), KW- Kernel width (mm), length to width ratio, VER-Volume expansion ratio, KLAC- Kernel length after cooking (mm), ER-Elongation ratio, ASV-Alkali Spreading Value, AC-Amylose content (%), GC-Gel consistency (mm), VOC-Very Occasionally Present, OC-Occasionally present, MS- Medium Slender, MB-Medium Bold

All the introgression lines displayed MS grain type and cooking qualities similar to that of ISM, except RP6287-178 which is medium bold

### Performance of DRR Dhan 58 in Station Trials and Multi-Location Trials

Based on the agro-morphological data, these same lines were evaluated as part of a yield trial conducted at ICAR-Indian Institute of Rice Research (ICAR-IIRR), Hyderabad during wet season (June to November) 2018 under normal conditions (Additional file 4: Table S3). Among them, three Improved lines (RP6287-43, RP6287-88 and RP6287-178) performed well and RP6287-88 (DRR Dhan 58), out yielded (5770 kg/ha) ISM (4713 kg/ha). Based on the yield performance in the station trial data and Na^+^ and K^+^ estimation in the same season, this entry was nominated for multi-location evaluation.

Comparative zonal data results for the nominated entry, DRR Dhan 58 (IET 28784), the recurrent parent ISM under normal and saline stress prone regions across India revealed that DRR Dhan 58 had superior yield performance (> 15%) over the recurrent parent in the two years of testing. The entry showed yield advantage under coastal salinity conditions (> 24% yield advantage) over the recurrent parent, (Progress Report, Vol. I, Varietal Improvement, AICRIP, ICAR-IIRR, 2019, 2020), 4.65% over the tolerant check (CSR10), and 46.86% over the sensitive check (Pusa 44) in Zones III, V and VII (Additional file 5: Table S5; Additional file 1: Figure S5). Based on its superior performance in both normal soils and coastal saline soils along with high levels of bacterial blight resistance and grain quality similar to ISM, DRR Dhan 58 has been released and identified as a new variety, by the Central Sub-Committee on Crop Standards, Notification and Release of Varieties for Agricultural Crops, Ministry of Agriculture, Govt. of India.

### Genomics-Based Background Genome Recovery Analysis of DRR Dhan 58

**Fig. 3:**
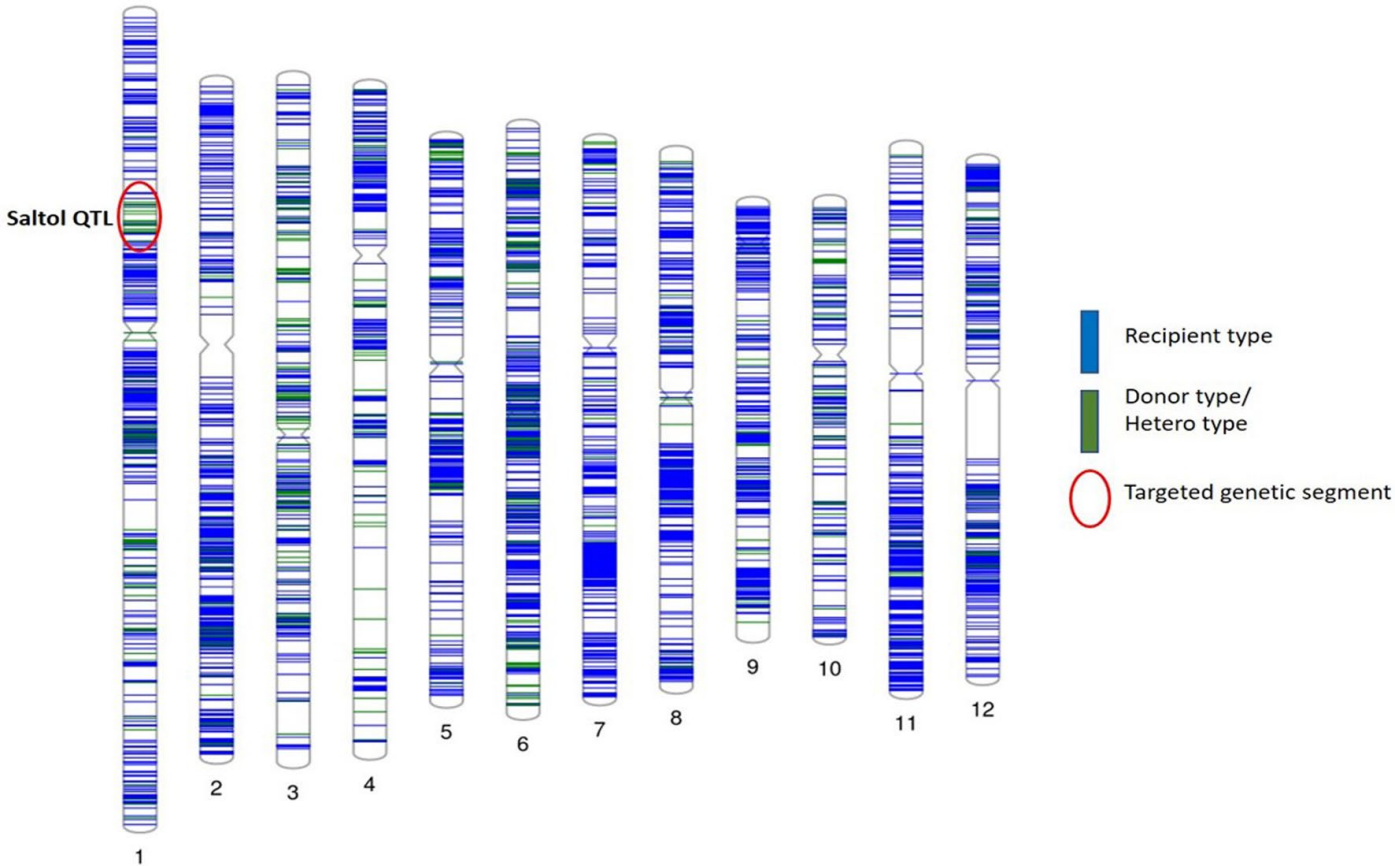
Graphical representation of Recurrent Parent Genome similarity of *Saltol* QTL with the ISM using 90 K SNP

The genetic background of DRR Dhan 58, FL478 and ISM were compared using the 90 K SNP chip. The results showed of 95.1% recovery of the ISM recurrent parent genome (Fig. 3).

### Assessment of Introgression of the Genome Region Around Saltol QTL in DRR Dhan 58

**Fig. 4:**
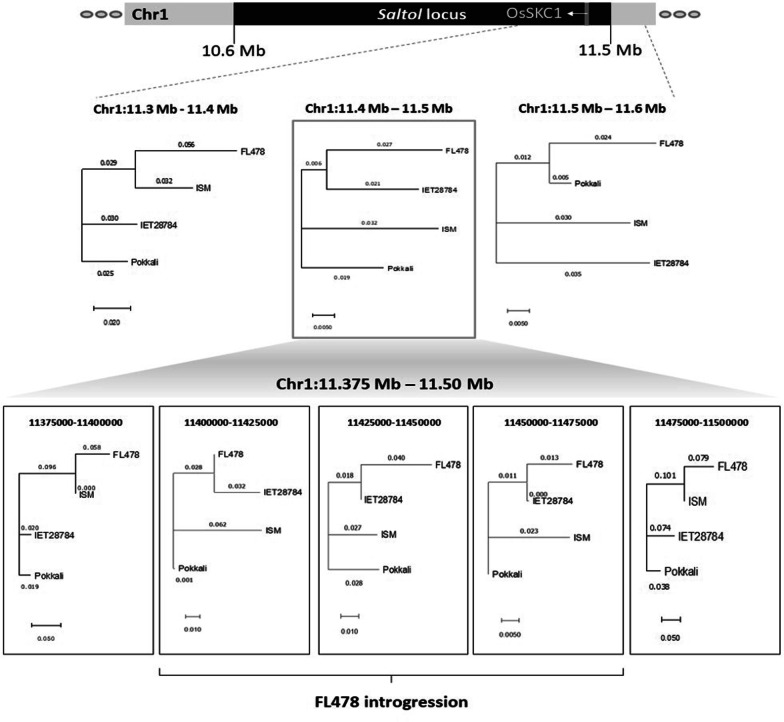
Analysis of 100 kb bins within the *Saltol* locus shows a relatedness between FL478 and DRR Dhan58 between 11.4 and 11.5 Mb interval (grey-boxed dendrogram). Further binning and subsequent analyses revealed the introgression of region between 11.4 and 11.475 Mb from FL478 into DRR Dhan58. The location of *OsSKC1* is highlighted as a grey line in the *Saltol* locus schematic. The numbers on the branches indicate the distance between the rice lines (IET 28784 is the line DRR Dhan 58)

In order to validate the introgression of *Saltol* locus in DRR Dhan 58, whole genome sequences of Pokkali, FL478, ISM, and DRR Dhan 58 were analyzed. The sequence and variants statistics are provided in the supplementary information (Additional files 6, 7, 8: Tables S6-S8). The results indicated no significant difference in the sequences among the tested rice lines within the *Saltol* locus (Chr1:10.6–11.5 Mb). However, at the *OsSKC1* locus (also known as *OsHKT1*;5), which was proposed to be the candidate gene responsible for seedling stage salt tolerance in the *Saltol* locus (Quan et al. 2018), the salt tolerant lines i.e., Pokkali, FL478 and DRR Dhan 58 showed a high sequence similarity (Additional file 1: Figure S6). This observation supports the hypothesis that the favorable *OsSKC1* allele in DRR Dhan 58 originated from Pokkali and was introgressed into FL478, the donor parent. There was a high level of relatedness between FL478 and DRR Dhan 58 in the region between 11.4 and 11.5 Mb of the *Saltol* locus. Further analysis within this bin showed the contribution of FL478 to DRR Dhan 58 genome between 11.40 and 11.475 Mb in the Chr1 *Saltol* locus. Notably, this interval encompasses the *OsSKC1* gene (11.458–11.463 Mb) (Fig. 4).

## Discussion

This study reports the incorporation of the major QTL, *Saltol,* conferring seedling stage salinity tolerance, into the elite, high-yielding, fine-grain type, bacterial blight resistant and low GI rice variety, ISM and validates the utility of the *Saltol* containing lines through multi-location trials conducted across India (Additional file 5: Table S5). Previously, the *Saltol* QTL was introgressed into the very popular, export quality Indian Basmati variety, Pusa Basmati 1509 (Ashutosh et al. 2020); four popular Vietnamese varieties, AS996, BT7, Bacthom 7 and Q5DB (Luu 2012; Linh et al. 2012; Vu et al. 2012; Soda et al. 2013) and the popular Bangladeshi variety, BRRI Dhan-49 (Hoque et al. 2015). These studies confirmed the lines with the *Saltol* QTL and showed excellent tolerance to seedling stage salinity.

In the present study, foreground selection was carried out using a set of five markers spanning *Saltol* QTL similar to the reports of Vu et al. (2012) and Huyen et al. (2013). Breeding lines were genotyped at each backcross generation with all the five markers and the lines with positive alleles for *Saltol* QTL were identified for further advancement. Stringent background selection was employed with SSR markers across the genome and backcross derived lines with maximum recurrent parent genome of ~ 95.3% was recovered at BC_3_F_2_ generation (Additional file 1: Figure S2). Our results are in agreement with Vu et al. (2012) and Huyen et al. (2013) who reported around 96% recurrent parent genome recovery. With three backcross generations the desirable attributes of the ISM recurrent parent were recovered, namely high yield, bacterial blight resistance, medium-slender grain type and premium grain quality. Near-complete recovery of the recurrent parent genome in the vicinity of target genes/QTL has been reported by Sanchez et al. (2000), Singh et al. (2001), Dokku et al. (2013), Suh et al. (2013) and Pradhan et al. (2015) for the bacterial blight resistance genes, *Xa4, xa5, xa13* and *Xa21*; by Gouda et al. (2013) for the blast resistance genes, *Pi*-*1* and *Piz-5*, by Kumar et al. (2014) for multiple QTLs associated with yield under drought. In this study, SNP genotyping of the *Saltol* region confirmed that DDR Dhan 58 (RP6287-88) only had a 2.5 Mb segment introgressed from the donor (FL478) based on SSR markers (Additional file 1: Figure S2).

Seedling stage salinity tolerant lines in the genetic background of ISM (i.e., possessing *Saltol*) were identified using the earlier developed protocol for rapid screening of seedling stage salinity tolerance (Rekha et al. 2018b). Utilizing this protocol, we were able to identify the seedling stage salinity tolerant lines of ISM (i.e., possessing *Saltol*) in an unambiguous manner as the tolerant lines (including the tolerant check, FL478) were clearly distinguished from the sensitive ones (including the ISM) (Additional file 1: Figure S3). The backcross derived lines of ISM possessing *Saltol* QTL, survived the stress with minor variations with respect to the degree of tolerance, with scores between 1 and 3 on a scale of 0–9, indicating a moderate to high level of tolerance, while the recurrent parent, ISM was scored as sensitive with a score of 9 and showing complete mortality. Among the identified lines, four NILs showed tolerance to salinity at very high salt concentration of 12 dS/m, similar to earlier reports of Bhowmik et al. (2007), Titov et al. (2009) and Mondal and Borromeo (2016) who identified elite lines possessing *Saltol* QTL conferring seedling stage salinity tolerance. The four NILs of ISM, RP6287-12, RP6287-43, RP6287-88 and RP6287-178 possessed a lower Na/K ratio similar to the donor parent (Additional file 3: Table S2A); suggesting the *Saltol* QTL possibly confers ion exclusion mechanism, mainly by reducing the Na^+^ concentration and increasing concentration of K^+^, resulting in tolerance to seedling stage salinity stress. This observation also suggests that the homeostasis between Na^+^ and K^+^ concentrations could be a key factor in conferring tolerance. Earlier, Chakraborty et al. (2020) reported the major mechanism of Na ion exclusion of FL478 is due to xylem unloading of the Na ion by hyper action of HKT1;5 transporters present in the nearby xylem parenchyma cells, which in turn contributes for tolerance. Similar results were also observed in our study (Additional file 3: Table S2A). Low salt injury score (i.e., IRRI-SES scores) coupled with low Na^+^ uptake and low Na/K ratio are the major contributing factors for salinity tolerance conferred by *Saltol* QTL, especially at seedling stage (Lisa et al. 2004). Identical observations viz., role of low Na^+^ uptake and low Na/K ratios were also reported by Puram et al. (2017) and Babu et al. (2017) in the donor, FL478, which possesses *Saltol*.

Under salinized conditions, reduced root length and its elongation was observed in sensitive rice varieties with increasing concentration of salt (Bhutta et al. 2004). Leaf and root Na content were also reported to be significantly increased in the presence of salt, while K content decreased, thus imposing a negative effect on root and shoot K/Na ratio (Kafi et al. 2011). In the present study, we observed that root length had a highly significant and positive correlation with low Na^+^/ K^+^ ratio indicating role of root length in better accumulation of Na^+^ and K^+^ ions under stress conditions (Additional file 3: Tables S2A and S2B; Fig. 2A and B). Similar results were observed by Lang et al. (2017) and Eti et al. (2018), wherein greater root length was identified to be a promising trait with respect to salinity tolerance. Since *Saltol* QTL is associated with Na^+^/K^+^ balance in the shoot tissues, the implicit mechanism of tolerance is attributed to Na^+^/K^+^ homeostasis driven by *OsHKT1*;5 (Singh et al. 2012). The *OsHKT1*;5 gene, also known as *SKC1*, encodes a xylem-expressed Na^+^ transporter, which is highly selective in upward Na ion transportation mechanism in FL478 and can unload Na ion from xylem stream, hence preventing upward transport of Na ion (Chakraborty et al. 2020). However, we did not observe any such difference between the cation content under unstressed conditions, indicating that ion homeostasis mechanisms might possibly be active only under salt stress.

Phenotypic screening using a virulent isolate of bacterial blight pathogen confirmed the earlier findings that the recurrent parent, ISM possesses high levels of resistance against bacterial blight disease (Rekha et al. 2018a). In fact, during the development of DDR Dhan 58, we selected lines with the genetic background of ISM, showing a high degree of bacterial blight resistance with a lesion length of 0.3 to 1.7 cm against *Xoo* with a score of 0–3, while FL478 showed susceptible reaction to bacterial blight disease, similar to previous reports (Rajpurohit et al. 2011; Dokku et al. 2013; Suh et al. 2013; Pradhan et al. 2015). All the selected lines possessed yield levels equivalent to or better than that of the recurrent parent ISM, along with medium-slender grain type and cooking quality characters.

Genetic enhancement of Samba Mahsuri in terms of bacterial blight resistance in addition to seedling stage salinity tolerance, while retaining the desirable grain and cooking qualities is indeed one of the major achievements of the present study. The developed line, DRR Dhan 58 also showed equivalent or superior performance over ISM at multiple locations in AICRIP trials and had a high level of bacterial blight resistance at all the test locations. Due to its superior performance with respect to salinity tolerance and bacterial blight resistance, DRR Dhan 58 was identified and released by Central Sub-Committee on Crop Standards, Notification and Release of Varieties for Agricultural Crops, Ministry of Agriculture, Government of India.

In this study, we used whole genome sequencing (WGS) to understand the introgression of regions from FL478 into DRR Dhan 58 specifically focusing on the *Saltol* locus. Our analyses clearly demonstrated the zone of introgression from FL478 into DRR Dhan 58 to be the ~ 75 kb interval between 11.4 and 11.475 Mb of the *Saltol* locus. As expected, regions flanking the FL478 introgression in DRR Dhan 58 were found to be highly related to the recurrent parent, ISM. Interestingly, the presence of *OsSKC1* in this region along with previous reports (Thomson et al. 2010 and Ashutosh et al. 2020) strongly indicates the role of this gene in regulating salt stress response thereby leading to tolerance. This observation further strengthens the possible role of *OsSKC1* in salt tolerance. Overall, SNP genotyping and WGS analysis revealed complete introgression of the *Saltol* locus in DRR Dhan 58 and near complete recovery of the genome of ISM (Additional file 1: Figure S6). In addition to DRR Dhan 58, which has been released for cultivation by farmers, the other three multi-trait pyramided lines of ISM possessing *Saltol*, developed through our study can serve as elite donors for resistance/tolerance to bacterial blight and salinity with good grain quality. Presently, we are analyzing the glycaemic index of DRR Dhan 58 through physical tests and clinical trials to validate the low GI trait of its grains.

## Conclusion

In the present study, we developed DRR Dhan 58, a salinity tolerant NIL of the elite rice variety, ISM through marker-assisted backcross breeding (MABB) strategy and validated the utilization of DRR Dhan 58 in multilocation evaluation. DRR Dhan 58 and the backcross inbred lines of ISM possessing salinity tolerance can serve as valuable resource for genetic improvement of rice in multiple stress tolerance breeding programme and genomic studies.

## Materials and Methods

### Plant Materials

ISM (RPBio-226), possessing three major bacterial blight resistant genes, identified as *Xa21, xa13* and *xa5*; developed through marker-assisted backcross breeding (Sundaram et al. 2008) was used as the recurrent parent, while FL478 (IR 66,946-3R-178-1-1; IRGC 117,406) possessing *Saltol,* major QTL for seedling stage salinity tolerance, (Thomson et al. 2010) was used as the donor parent. Apart from these, Taichung Native1 (RDA-Gene bank Information Centre accession no. IT004120) and ISM were used as susceptible and resistant checks, respectively for bacterial blight screening; while FL478 and ISM were used as tolerant and sensitive checks, respectively for seedling stage salinity screening.

### MABB Method for Developing DRR Dhan 58

The marker-assisted backcross breeding (MABB) approach was deployed to introgress the *Saltol* QTL locus into the genetic background of the recurrent parent, ISM (Additional file 1: Figure S1). Genomic DNA extraction from the leaf tissues, collected from the parental lines and the backcross derived lines was carried out using modified CTAB method as explained in Sundaram et al. (2009). PCR amplification, gel electrophoresis and visualization of markers specific amplicons for target genes/QTL as per Rekha et al. (2018a) with respect to the bacterial blight resistance genes and as per Sundaram et al. (2009) for the SSR markers specific for *Saltol* QTL. The genome-wide polymorphism survey was carried out with a total of 200 SSR markers selected from Gramene (https://archive.gramene.org/markers/microsat/all-ssr.tab) and uniformly distributed across all 12 rice chromosomes. From these markers, a set of 66 parental polymorphic hyper-variable SSR markers, which were distributed across the rice genome (Additional file 2: Table S1) were used for background selection.

The development of DRR Dhan 58 is diagrammed in Additional file 1: Fig. S1. Briefly, true F_1_s were identified using the SSR marker, RM3412, which is the closest marker for *Saltol* QTL (Thomson et al. 2010). These F_1_s were then backcrossed to the recurrent parent and a single BC_1_F_1_ plant was identified that was heterozygous for the five SSR markers, specific for *Saltol* locus, RM1287, RM8094, RM3412, RM493 and RM140, spanning the *Saltol* QTL region from 10.6 Mb to 11.6 Mb (Thomson et al. 2010) and homozygous for the three bacterial blight resistance genes, *Xa21* (identified by pTA248; Ronald et al. 1992), *xa13* (identified by xa13prom; Hajira et al. 2016) and *xa5* (identified by xa5FM; Hajira et al. 2016), and possessing maximum recurrent parent genome recovery based on 66 background markers having the ISM allele as explained in Sundaram et al. (2008). The process of marker-assisted foreground selection for *Saltol* using the five SSR markers mentioned above and background selection with 66 polymorphic SSR markers continued to BC_3_F_1_ generation, wherein a single plant, heterozygous for *Saltol* locus and possessing the bacterial blight resistance genes in homozygous condition with maximum recovery of ISM genome was selfed to generate BC_3_F_2_s. Among them, a single BC_3_F_2_ plant possessing the entire *Saltol* locus in homozygous condition and maximum recurrent parent genome was advanced to the BC_3_F_4_ generation based on phenotypic pedigree selection. Four promising BC_3_F_4_ lines (RP6287-12, RP6287-43, RP6287-88 and RP6287-178) possessing the *Saltol* QTL along with the three bacterial blight resistance genes and closely resembling ISM in terms of plant type and grain type were analysed for their resistance against bacterial blight and tolerance to seedling stage salinity and recurrent parent genome recovery among these four lines was summarized using the software Graphical genotyping (GGT) version 2.0 (Van Berloo 1999).

### Phenotypic Screening for Bacterial Blight Resistance

Among the gene-pyramid lines of ISM, four promising lines possessing *Saltol* QTL, three bacterial blight resistance genes *Xa21, xa13* and *xa5* along with the plant type and grain type similar to the recurrent parent, were screened at BC_3_F_4_ generation at their maximum tillering stage for their resistance to bacterial blight during dry season 2018 (December to April) using a local, virulent isolate *Xanthomonas oryzae* pv. *oryzae*, DX-020 (Laha et al. 2009) with a final concentration of 10^5^ cfu/ml (Preece, 1982) by clipping through clip inoculation method of Kauffman et al. (1973). Plant inoculation was done by clipping the leaf tip of the fully expanded uppermost leaf using scissors dipped in the inoculum (about 1 to 2 cm). Visible symptoms appear as water soaking on 4th day after inoculation and mean percentage of diseased leaf area (%DLA) was measured at 15 days after inoculation. The inoculated plants were scored using the standard IRRI -SES scale (IRRI, 2013), 15-days after inoculation. The rating scale score 1 was 5% of leaf area diseased; score 3 was 6–12% of leaf area diseased; score 5 was 13–25% of the leaf area diseased; score 7 was 26–50% of the leaf area diseased; score 9 was 51–100% of the leaf area diseased.

### Phenotypic Screening for Seedling Stage Salt Tolerance

Four ISM lines possessing *Saltol* at BC_3_F_4_ generation, along with the sensitive and tolerant checks were screened for the seedling stage tolerance under glass house conditions maintained at 27–30 °C/21–25 °C of day/night temperature, relative humidity of 70% under natural daylight at ICAR-IIRR during dry season 2018 (December to April). The experimental set up included the replacement of hydroponics with silica sand inert base (Three-fourth of the tray was filled with 2 mm size silica sand), then pre-germinated seedlings were transferred into trays filled with Yoshida medium (pH 5.5–6.0). After 21 days, salt stress treatment at 120 mM NaCl was imposed on the seedlings, as well as a negative control, lacking NaCl. Details of the experimental method are described in Rekha et al. (2018b). Entries were scored based on the visual symptoms using IRRI standard scale (IRRI, 2013). Apart from the visual phenotypic scoring, shoot length and root length, fresh and dry weight of shoot and root, Na^+^ and K^+^ ratio of both control and treated samples also were considered. To measure shoot length and root length, three randomly selected uniform looking plants for each line within each replication, were carefully collected from both the treated and untreated control trays. Shoot length was measured from base of the culm to the tip of the tallest leaf and each whole plant was washed 2 to 3 times with deionized water and the length from the crown of the root to the root tip was measured in centimetres. The plants were then cut at the collar region to separate root and shoot portions. For each plant, fresh weight of shoot and root portion were immediately recorded and dried in a hot air oven at 60 °C and the dry weights were recorded. These dried samples were used for the estimation of Na and K content in these tissues as per the procedure described by Babu et al. (2017).

### Evaluation of Agronomic and Grain Quality Traits

Four selected improved lines of ISM, possessing *Saltol* at BC_3_F_5_ generation, were transplanted in the experimental farm of ICAR-IIRR during wet season 2018 (i.e., June to November) along with the recurrent parent, ISM at a spacing of 20 × 15 cm in three replications in plots, each of 1m^2^ area following randomized block design (RBD) with a salinity regime of 2 dS/m (non-saline conditions). The plants were raised by adopting a standard package of practices till their maturity. Data was recorded among 33 plants for each replication (n = 3) for the agro-morphological traits, days to 50% flowering (DFF), plant height (cm), number of productive tillers per plant, panicle length which was measured from the neck of the node i.e., panicle base to the end of the panicle (cm), grain yield per 33 plants (i.e., per m^2^; gm), 1000-grain weight (gm) as explained in Sundaram et al. (2008) and Hari et al. (2013). The grain and cooking quality features of the harvested grains from improved lines and the respective parents were assessed for the traits like hulling, milling, kernel length and breadth, grain chalkiness, water content, volume expansion ratio (VER), kernel length after cooking (KLAC), elongation ratio, alkali spreading value, amylose content and gel consistency were also recorded as explained in Sundaram et al. (2008).

### Evaluation of DRR Dhan58 Under Multilocation Tests (AICRIP)

Four NILs of ISM (RP6287-12, RP6287-43, RP6287-88 and RP6287-178) possessing the bacterial blight resistance genes (*Xa21, xa13* and *xa5*) and salinity tolerance QTL, *Saltol* were selected based on the agro-morphological data and station trial data conducted at ICAR-IIRR, Hyderabad. The entry, RP6287-88 was selected based on its superior performance over ISM in terms of yield and nominated in AVT-NIL-CS (Advanced varietal trial-Near Isogenic lines-coastal salinity) for evaluation along with recurrent parent, ISM. Site characterization for the tested locations is reported in Additional file 9: Table S4.

### Statistical Analysis

A two-way analysis of variance (ANOVA) was used to determine significant differences for different agro-morphological parameters using SAS Version 9.2 (SAS Institute Inc., Cary, NC, USA) software program; PROC GLM procedure of SAS was used to conduct ANOVA to determine the significant variation between the lines. Critical Difference (CD), Coefficient of variance (CV), at *p* = 0.05 were calculated using standard errors of mean (S. E. M. ±) using MS Excel package.

$$\sigma {\text{M}} = \sigma /\surd {\text{N}}$$

*σ*M = Standard error of mean.

*σ* = Standard deviation of original distribution.

√N = Square root of the sample size.

Correlation among the phenotypic screening data under stressed and unstressed conditions were analyzed using PERFORMANCE ANALYTICS Package in the statistical software ‘R’ language (R Core Team 2016).

### Analysis of Recurrent Parent Genome Recovery Using 90K SNP

To assess the background recovery of the recurrent parent, DRR Dhan58 was compared with recurrent parent ISM using Affymetrix 90 K Axiom® 2.0 SNP chip ‘OsSNPnks’ on GeneTitan® instrument (IARI, unpublished). Genomic DNA amplification, fragmentation, chip hybridization, washing, single-base extension through DNA ligation, and signal amplification was carried out according to Affymetrix Axiom® 2.0 Assay Manual Target Prep Protocol. Staining, washing, and scanning were performed using GeneTitan integrated platform (http://www.affymetrix.com). SNP genotypes were called using the Affymetrix Genotyping Console™ v4.1 (AGC) software package. SNPs with low call rates across all samples were removed from the dataset and high-performing SNPs with a development quality check (DQC) score of > 0.85, and call rates of > 95.0% were used for further analyses (Singh et al. 2015). Graphical representation of 90 K SNP genotyping based recurrent parent genome recovery was done using Phenogram software from Ritchie lab, Penn. State University, Pennsylvania, USA (http://visualization.ritchielab.psu.edu/ phenograms/plot). The recurrent parent genome recovery similarity based on SNP markers was calculated using the formula (Ellur et al. 2015).

$${\text{Recurrent}}\;{\text{Parent}}\;{\text{Genome}}\;\left( {{\text{RPG}}} \right)\left( \% \right)\, = \,\left( {{\text{R}}\, + \,{1}/{\text{2H}}} \right)\, \times \,{1}00/{\text{P}},$$

where R = number of markers homozygous for RP allele,

H = number of heterozygous markers,

P = total number of SNP markers used for background selection.

### Whole Genome Resequencing and Analysis

Genomic DNA from the seedlings of Pokkali, ISM and DRR Dhan58 was isolated using the CTAB method (Saghai-Maroof 1984). Quality and quantity of the DNA samples were assessed using agarose gel electrophoresis and Qubit HS dsDNA assay kit (Invitrogen, USA), respectively. DNA library was prepared using Illumina DNA Prep kit (Illumina, USA) with a target insert size of ~ 350 bp. The libraries were sequenced using Illumina’s NovaSeq6000 to obtain 150 bp paired-end reads.

Quality and statistics of the sequenced reads were assessed using FastQC (v0.11.8). FL478 sequence from NCBI SRA was used for the comparative analysis (Accession: SRR9943932). All the reads were aligned to the Nipponbare reference genome (MSU version 7) using bwa-mem (v0.7.17-r1188; http://bio-bwa.sourceforge.net/bwa.shtml). The alignment files were sorted and deduplicated using samtools (v1.7; http://www.htslib.org/doc/samtools.html). Variants were called and filtered using bcftools mpileup and filter commands (v1.7; http://samtools.github.io/bcftools/bcftools.html). Variants were filtered based on the criteria including minimum mapping quality of 40, minimum base quality of 20, minimum and maximum depth of 10 and 200, respectively, for Pokkali, ISM, and DRR Dhan58, and 5 and 200, respectively, for FL478. Variants extraction from specific genomic intervals was performed using bed tools intersect option (v2.26.0; https://bedtools.readthedocs.io/en/latest/). Statistics on genome coverage, and sequence depth were obtained using in house bash scripts. Variant statistics were obtained using bcftools stats option. SnpEff (v4.3t; Cingolani et al. 2012) was used to annotate the variants. Tassel 5 was used to study the relatedness between the rice lines and obtain the neighbor-joining tree based on the variants data (Bradbury et al. 2007). The relatedness dendrograms were visualized using MEGA X (Sudhir Kumar et al. 2018).

## Supplementary Information

**Additional file 1**. **Figure S1**: Marker assisted backcross breeding strategy used in the present study. **Figure S2**: Analysis of donor parent genome introgression associated with Salinity tolerance QTL, Saltol locus using GGT version (2.0), a segment of 1.0 Mb was introgressed at the proximal end, 1.5Mb at the distal end from the donor parent genome in the best BC3F2 plant (i.e., RP6287-188-45-12-88), thus, in total, a segment of 2.5Mb was introgressed from the donor parent with respect to the genomic region in the vicinity of Saltol. The position of the polymorphic SSR markers in Mb on Chr. 1 is given in parenthesis adjacent to each marker. **Figure S3**: Screening for seedling stage salinity tolerance. (A) Uprooted seedlings of checks (ISM and FL478) and improved lines of ISM possessing Saltol QTL (IL1 to IL4), (B) Root morphology of checks ISM and FL478 along with introgressed lines IL-1(RP6287-88), IL-2 (RP6287-43), IL-3 (RP6287-12) and IL-4 (RP6287-178). **Figure S4**: (A): Improved bacterial blight and Salinity tolerance lines of ISM (B): Improved Salinity tolerance lines with better grain and panicle number; IL-1(RP6287-88), IL-2 (RP6287-43), IL-3 (RP6287-12) -Introgressed lines of ISM. **Figure S5**: Map depicting the salinity effected areas in the coastal regions of India (AICRIP centres). Map depicts the centres -Zone wise where the AICRIP trials were conducted and each zone was indicated in different colours. **Figure S6**: Frequency of the variants (SNPs, Insertions, and Deletions) per Mb that are present in the rice lines including Pokkali, FL478, ISM, and DRR Dhan 58 (IET28784). **Figure S7**: Neighbor-joining dendrograms showing the relatedness among the rice lines at the (A) Saltol locus (~0.9 Mb interval) and (B) the OsSKC1 gene based on the variants data. The data indicates no considerable difference in the relatedness among the rice lines ta the Saltol locus but a clear relatedness among the salt-tolerant lines at the OsSKC1 gene locus. The numbers on the branches indicate the distance between the rice lines (DRR Dhan58 is the line IET28784).

**Additional file 2**. **Table S1**: List of SSR markers used for background analysis.

**Additional file 3**. **Table S2A**: Estimation of Na and K in the introgressed lines of ISM with respect to recurrent and donor parents (stressed). **Table S2B**: Estimation of Na and K in the introgressed lines of ISM with respect to recurrent and donor parents (unstressed).

**Additional file 4**. **Table S3**: Station trial data of promising entries during wet season 2018 (June to Nov).

**Additional file 5**. **Table S4**: Site characterization of tested location data during Kharif 2019 and 2020

**Additional file 6**. **Table S6**: Sequencing and mapping statistics of the rice lines used in this study with respect to the Nipponbare reference genome

**Additional file 7**. **Table S7**: Distribution of variants (SNPs, Insertions and Deletions) across the chromosomes in the rice lines including Pokkali, FL478, ISM, and DRR Dhan 58.

**Additional file 8**: **Table S8**: Distribution of variants based on the location in the genome of the rice lines and their consequences.

**Additional file 9**. **Table S5**: Summary of yield (kg/ha) data of Zone wise Advanced varietal trails (AVT NIL) for Kharif 2019 and 2020 (June to Nov)

## Data Availability

The raw DNA sequence reads generated for this study are deposited in NCBI Sequence Reads Archive (SRA) under the BioProject ID PRJNA757580. The information about the variety, DRR Dhan 58 is available in the minutes of meeting of 87th meeting of Central Sub-Committee on Crop Standards, Notification and Release of Varieties for Agricultural Crops conducted by Ministry of Agriculture, Govt. of India on 22 September 2021and 18 October 2021. The SNP analysis was carried out through a commercially available SNP chipset and can be shared based on request.

## Abbreviations

MABB: Marker-assisted backcross breeding
*Xoo*: *Xanthomonas oryzae* Pv. *oryzae*
QTL: Quantitative trait loci
NIL: Near-isogenic line
GGT: Graphical genotyping
DNA: Deoxyribonucleic acid
CTAB: Cetyl trimethy ammonium bromide
SSR: Simple sequence repeats
ICAR-IIRR: Indian Council of Agriculture Research- Indian Institute of Rice Research
CSIR-CCMB: Council of Scientific and Industrial Research- Centre for Cellular and Molecular Biology
AICRIP: All India Coordinated Rice Improvement Project
