# Supplementary material for: DRR Dhan 58, a Seedling Stage Salinity Tolerant NIL of Improved Samba Mahsuri Shows Superior Performance in Multi-location Trials
Source: Rice (N Y). 2022 Aug 17;15:45. doi: 10.1186/s12284-022-00591-3 (PMC9385912; doi:10.1186/s12284-022-00591-3)
Supplement: Supplementary file 1 — Additional file 1. Figure S1: Marker assisted backcross breeding strategy used in the present study. Figure S2: Analysis of donor parent genome introgression associated with Salinity tolerance QTL, Saltol locus using GGT version (2.0), a segment of 1.0 Mb was introgressed at the proximal end, 1.5Mb at the distal end from the donor parent genome in the best BC3F2 plant (i.e., RP6287-188-45-12-88), thus, in total, a segment of 2.5Mb was introgressed from the donor parent with respect to the genomic region in the vicinity of Saltol. The position of the polymorphic SSR markers in Mb on Chr. 1 is given in parenthesis adjacent to each marker. Figure S3: Screening for seedling stage salinity tolerance. (A) Uprooted seedlings of checks (ISM and FL478) and improved lines of ISM possessing Saltol QTL (IL1 to IL4), (B) Root morphology of checks ISM and FL478 along with introgressed lines IL-1(RP6287-88), IL-2 (RP6287-43), IL-3 (RP6287-12) and IL-4 (RP6287-178). Figure S4: (A): Improved bacterial blight and Salinity tolerance lines of ISM (B): Improved Salinity tolerance lines with better grain and panicle number; IL-1(RP6287-88), IL-2 (RP6287-43), IL-3 (RP6287-12) -Introgressed lines of ISM. Figure S5: Map depicting the salinity effected areas in the coastal regions of India (AICRIP centres). Map depicts the centres -Zone wise where the AICRIP trials were conducted and each zone was indicated in different colours. Figure S6: Frequency of the variants (SNPs, Insertions, and Deletions) per Mb that are present in the rice lines including Pokkali, FL478, ISM, and DRR Dhan 58 (IET28784). Figure S7: Neighbor-joining dendrograms showing the relatedness among the rice lines at the (A) Saltol locus (~0.9 Mb interval) and (B) the OsSKC1 gene based on the variants data. The data indicates no considerable difference in the relatedness among the rice lines ta the Saltol locus but a clear relatedness among the salt-tolerant lines at the OsSKC1 gene locus. The numbers on the branches indicat [file 12284_2022_591_MOESM1_ESM.docx]

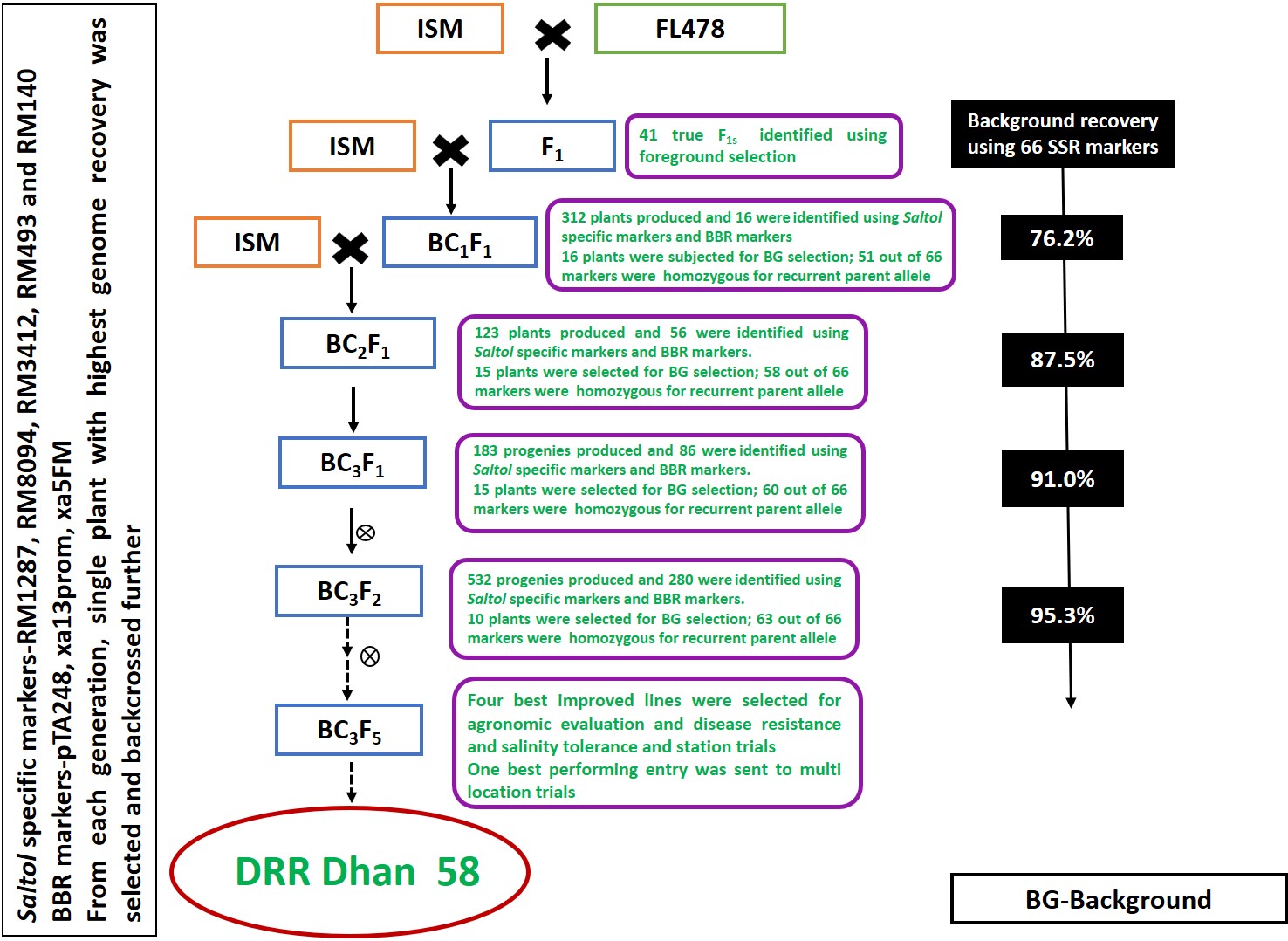


**Additional file 1: Fig. S1:** Marker assisted backcross breeding strategy used in the present study


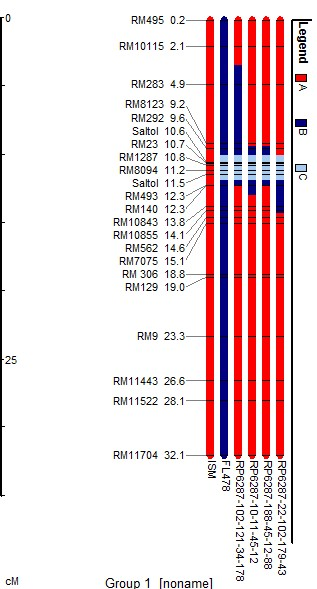


**Chromosome 1**

**Additional file 1: Fig. S2:** Analysis of donor parent genome introgression associated with Salinity tolerance (*Saltol*) QTL*,* *Saltol* locus using GGT version (2.0), a segment of 1.0 Mb was introgressed at the proximal end, 1.5Mb at the distal end from the donor parent genome in the best BC_3_F_2_ plant (i.e., RP6287-188-45-12-88), thus, in total, a segment of 2.5Mb was introgressed from the donor parent with respect to the genomic region in the vicinity of *Saltol.* The position of the polymorphic SSR markers in Mb on Chr. 1 is given in parenthesis adjacent to each marker.


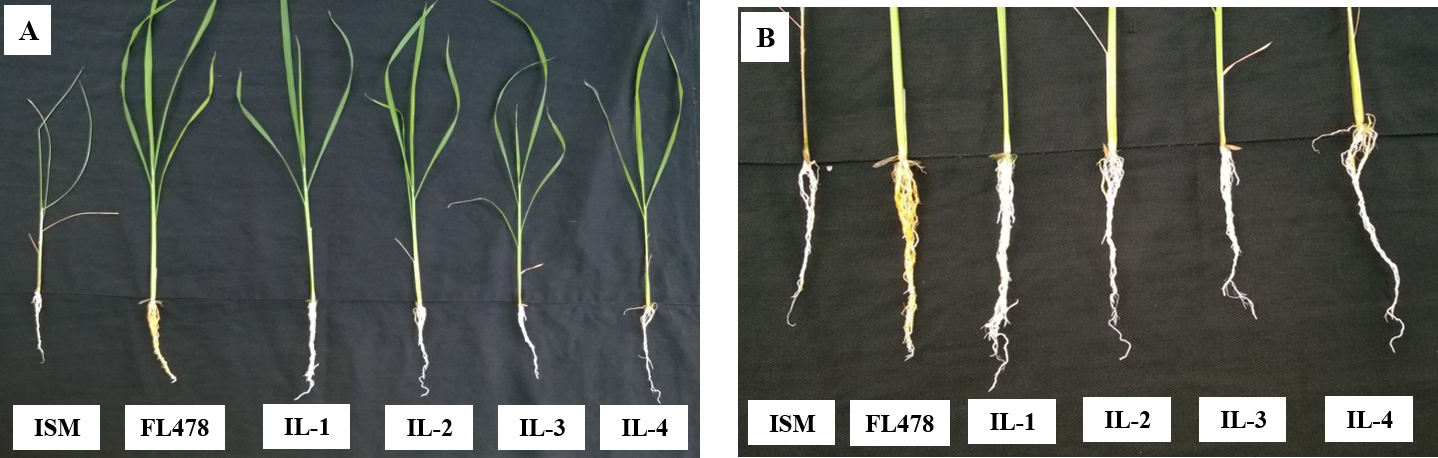


**Additional File 1: Fig. S3**: Screening for seedling stage salinity tolerance. **(A)** Uprooted seedlings of checks (ISM and FL478) and improved lines of ISM possessing *Saltol* (IL1 to IL4), **(B)** Root morphology of checks ISM and FL478 along with introgressed lines IL-1 (RP6287-88), IL-2 (RP6287-43), IL-3 (RP6287-12) and IL-4 (RP6287-178).


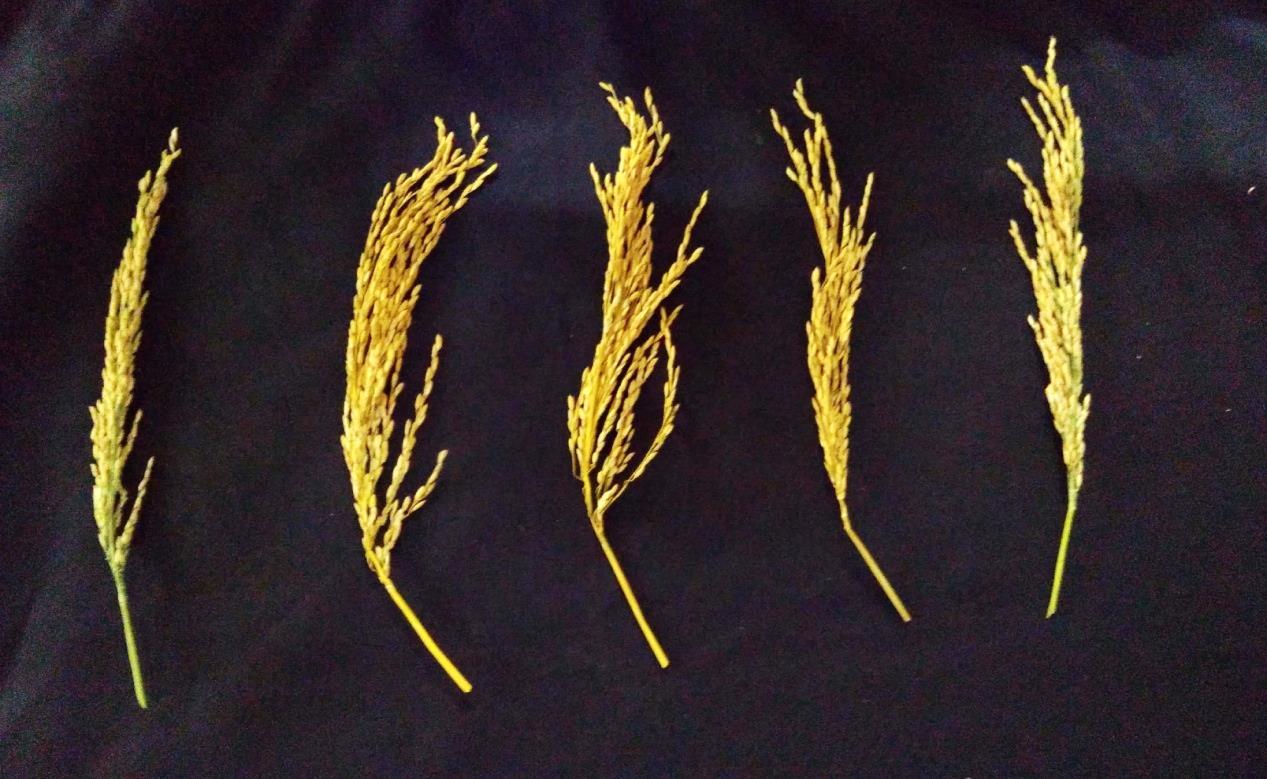


**ISM IL-1 IL-2 IL-3**


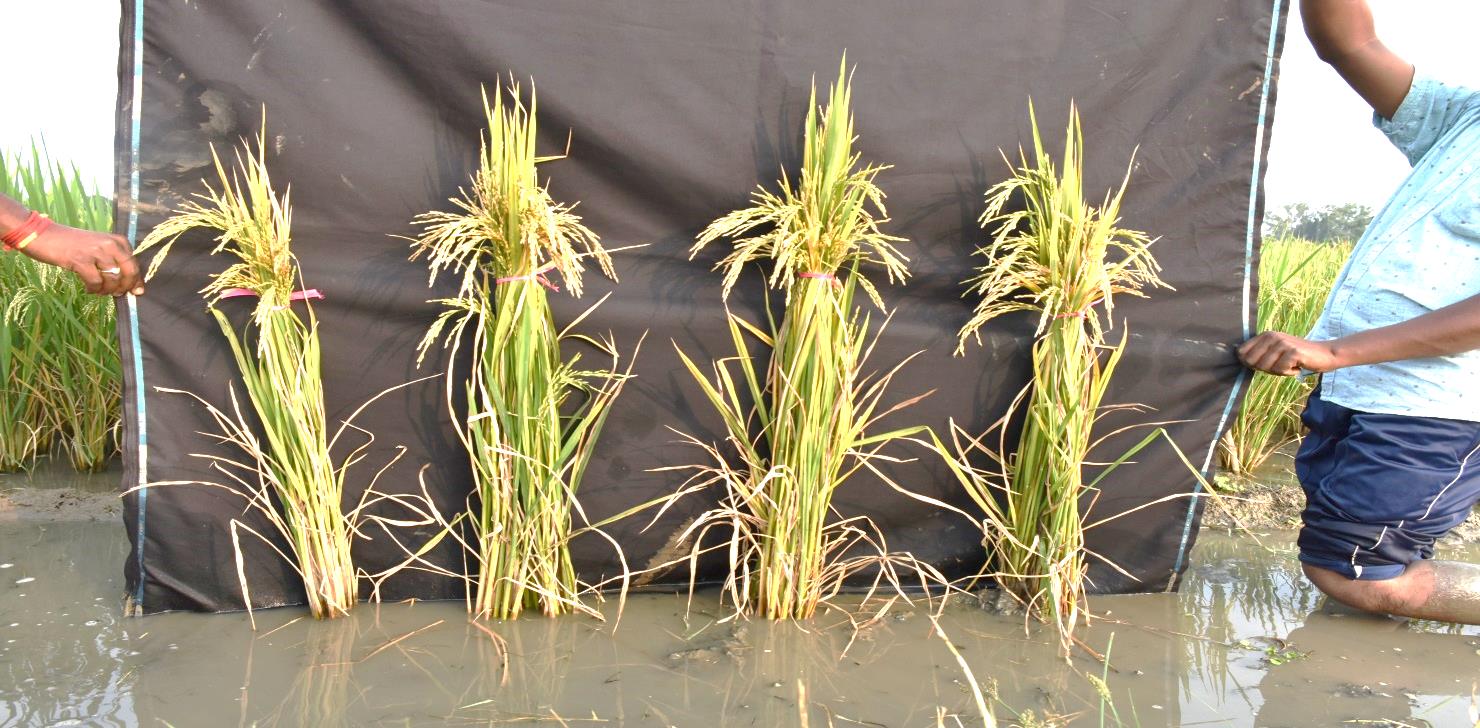


**ISM IL-1 IL-2 IL-3**

**A**

**B**

**Additional File 1: Fig. S4 (A)** Improved BB and Salinity tolerance lines of ISM (**B)** Improved Salinity tolerance lines with better grain and panicle number; IL-1 (RP6287-88), IL-2 (RP6287-43) and IL-3 (RP6287-12) – Introgressed lines of ISM

**
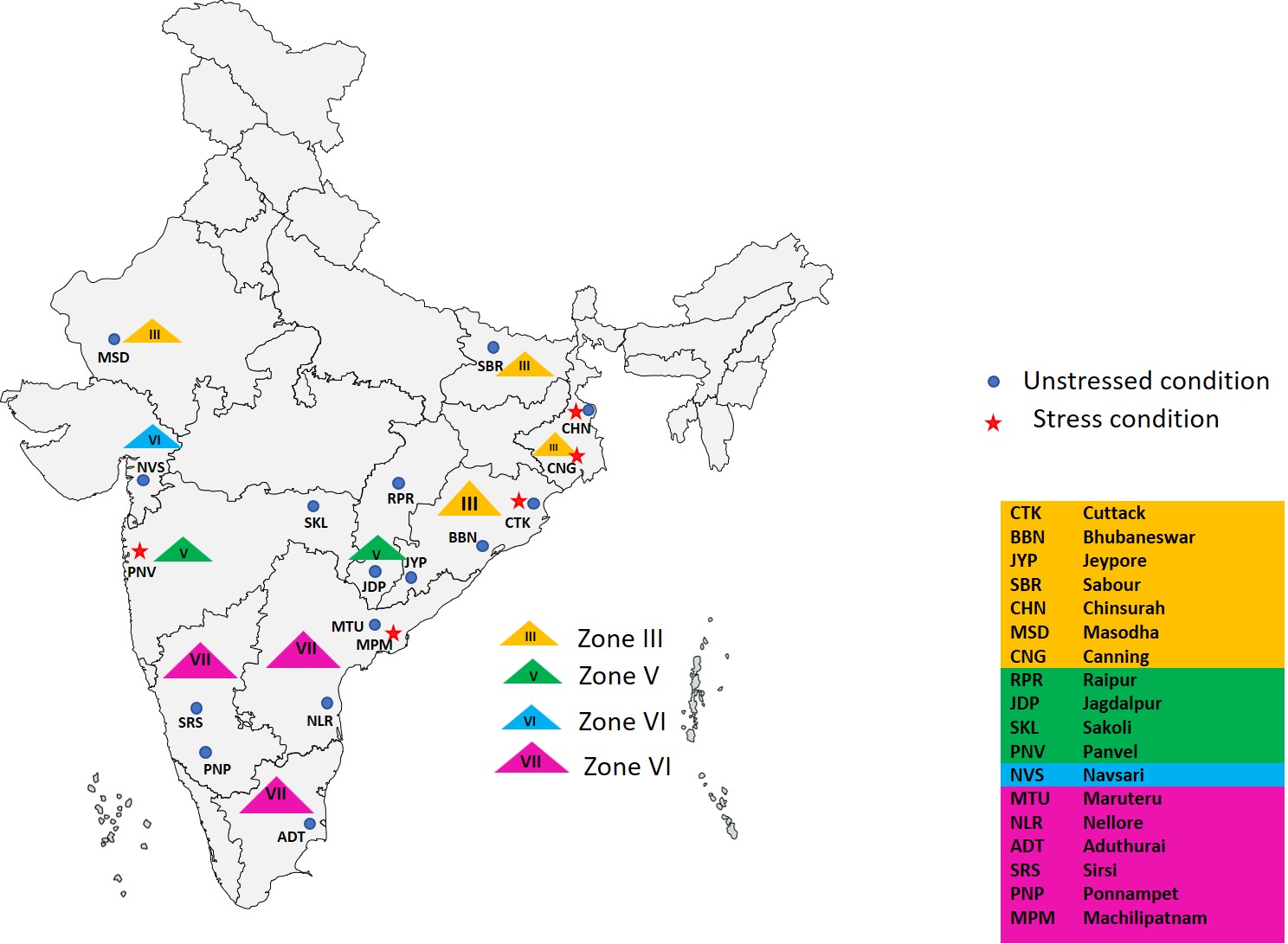
**

**Additional file 1 Fig. S5** Map depicting the salinity effected areas in the coastal regions of India (AICRIP centres). Each colour code represents each zone


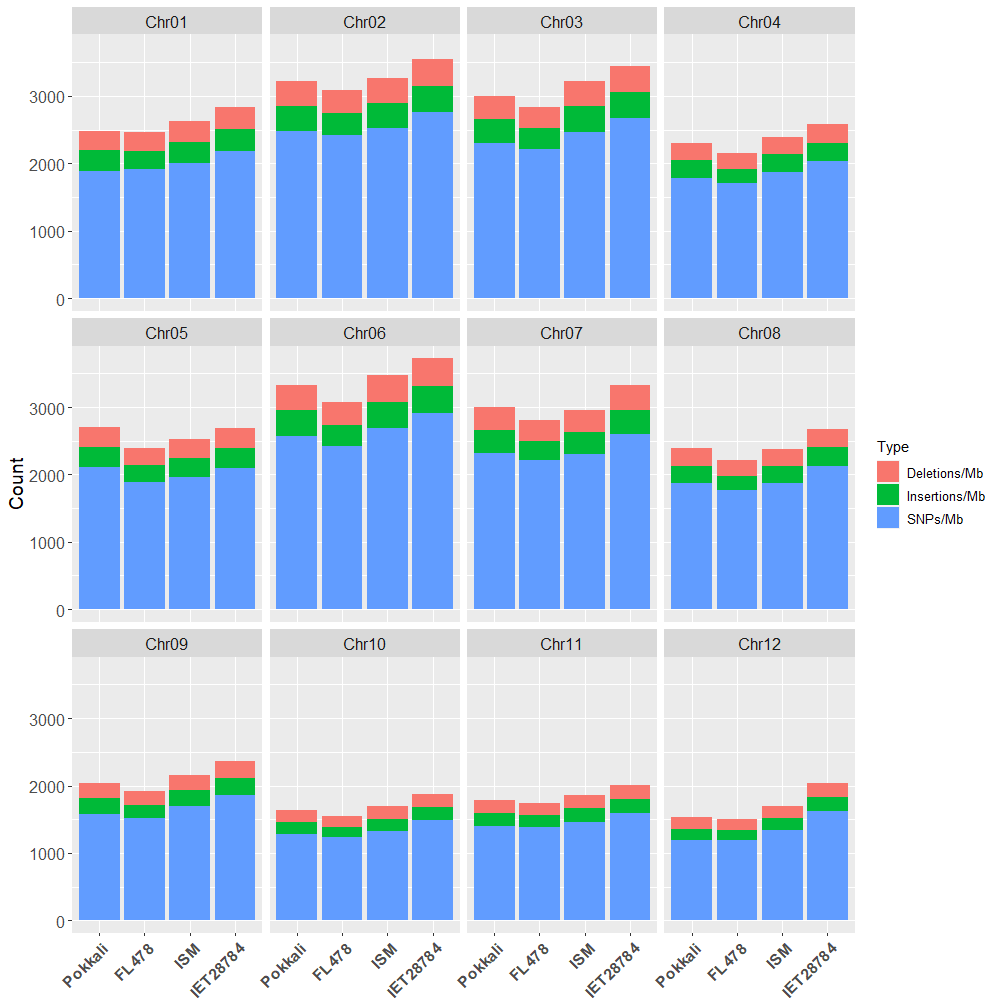


**Additional file 1 Fig. S6:** Frequency of the variants (SNPs, Insertions, and Deletions) per Mb that are present in the rice lines including Pokkali, FL478, ISM and DRR Dhan58 (IET28784).


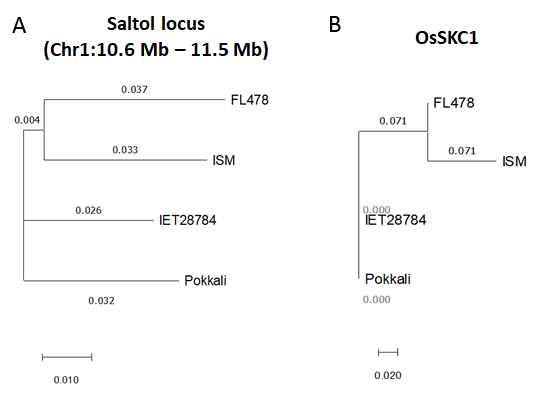


**Additional file 1 Fig. S7:** Neighbor-joining dendrograms showing the relatedness among the rice lines at the (A) *Saltol* locus (~0.9 Mb interval) and (B) the *OsSKC1* gene based on the variants data. The data indicates no considerable difference in the relatedness among the rice lines to the *Saltol* locus and a clear relatedness among the salt-tolerant lines at the *OsSKC1* gene locus. The numbers on the branches indicate the distance between the rice lines (DRR Dhan58 is the line IET28784).
