# Supplementary material for: DRR Dhan 58, a Seedling Stage Salinity Tolerant NIL of Improved Samba Mahsuri Shows Superior Performance in Multi-location Trials
Source: Rice (N Y). 2022 Aug 17;15:45. doi: 10.1186/s12284-022-00591-3 (PMC9385912; doi:10.1186/s12284-022-00591-3)
Supplement: Supplementary file 2 — Additional file 2. Table S1: List of SSR markers used for background analysis. [file 12284_2022_591_MOESM2_ESM.docx]

**Additional file 2: Table S1:** List of SSR markers used for background analysis

| **S. No** | **Primer name** | **Chr No.** | **Forward Primer Sequence** | **Reverse Primer Sequence** | **Physical position (Mb)** |
| --- | --- | --- | --- | --- | --- |
| 1 | RM495 | 1 | AATCCAAGGTGCAGAGATGG | CAACGATGACGAACACAACC | 0.21 |
| 2 | RM10115 | 1 | ACAAGACGAGGTAACACGCAAGC | GCGAAGGATCAACGATGATATGG | 2.1 |
| 3 | RM283 | 1 | GTCTACATGTACCCTTGTTGGG | CGGCATGAGAGTCTGTGATG | 4.88 |
| 4 | RM8123 | 1 | TATTCCGGTTTCATAATTCTTAATGTTTGG | GGGTTAAGGGTAGTTGTGCCTTATATTTTG | 9.23 |
| 5 | RM292 | 1 | ACTGCTGTTGCGAAACGC | TGCAGCAAATCAAGCTGGAA | 9.56 |
| 6 | RM8094 | 1 | AAGTTTGTACACATCGTATACA | CGCGACCAGTACTACTACTA | 11.23 |
| 7 | RM493 | 1 | TAGCTCCAACAGGATCGACC | GTACGTAAACGCGGAAGGTG | 12.28 |
| 8 | RM10843 | 1 | CACCTCTTCTGCCTCCTATCATGC | GTTTCTTCGCGAAATCGTGTGG | 13.7 |
| 9 | RM10855 | 1 | CCAAACAAGATGTAGTGGGAACATCC | CAAGATGATCAGTGGGCTATTCTTGG | 14 |
| 10 | RM7075 | 1 | TATGGACTGGAGCAAACCTC | GGCACAGCACCAATGTCTC | 15.11 |
| 11 | RM129 | 1 | TCTCTCCGGAGCCAAGGCGAGG | CGAGCCACGACGCGATGTACCC | 19 |
| 12 | RM9 | 1 | GGTGCCATTGTCGTCCTC | ACGGCCCTCATCACCTTC | 23.32 |
| 13 | RM423 | 2 | AGCACCCATGCCTTATGTTG | CCTTTTTCAGTAGCCCTCCC | 3.83 |
| 14 | RM424 | 2 | TTTGTGGCTCACCAGTTGAG | TGGCGCATTCATGTCATC | 11.38 |
| 15 | RM13154 | 2 | GGTACTTAGCGTGCAACTTTAACC | TAGGTAACTAGACGAAGCGATAGAGG | 15.22 |
| 16 | RM13761 | 2 | ATCCATCCTCAGACACACATGG | GAATTCCTGATATTCTCGCGTAGC | 27.5 |
| 17 | RM1342 | 2 | AGAAACCAAAGATGGGAGGG | CTAGCCAGCTCTCCCTTTTG | 28.15 |
| 18 | RM5686 | 3 | AACAGTGATGGTGGTGATGAATGG | GCTCAGGGACTGATCTGCTATGG | 8.54 |
| 19 | RM15561 | 3 | ATTAGCTTGGGCGTCTTCCTCTGG | TGCAAACAATGGCTTCACATCG | 24.63 |
| 20 | RM347 | 3 | CACCTCAAACTTTTAACCGCAC | TCCGGCAAGGGATACGGCGG | 30.73 |
| 21 | RM514 | 3 | CTTCTCAGATTGATCTCCCATTCC | GGGAGAGAGGAAGAAGACAAGG | 35 |
| 22 | RM16394 | 4 | CTCCACATTGTCTTTGGACTTGG | AAGACGTGGAGGAGAAGAAGAAGG | 3.39 |
| 23 | RM307 | 4 | GTACTACCGACCTACCGTTCAC | CTGCTATGCATGAACTGCTC | 11.11 |
| 24 | RM6997 | 4 | CGGCAGTAAATTTGCATTGACC | AGTGGCCTTGTCAGTCTACATGC | 21.28 |
| 25 | RM252 | 4 | TTCGCTGACGTGATAGGTTG | ATGACTTGATCCCGAGAACG | 24.02 |
| 26 | RM17448 | 4 | ATTTGCTAGCCAGGGTGCTAAGG | AGGGAGGGAGTGGCATAAATGG | 30.43 |
| 27 | RM17774 | 5 | TGTGGTAATCTCTAGGCCTTCTCC | ACCGACCAAAGATGGTTTCTCC | 0.6 |
| 28 | RM1386 | 5 | TCAAGCTGCATTAGGAAGACACC | AACTTAGCTGAAACGCAACACG | 19.9 |
| 29 | RM161 | 5 | TGCAGATGAGAAGCGGCGCCTC | TGTGTCATCAGACGGCGCTCCG | 20.9 |
| 30 | RM178 | 5 | TCGCGTGAAAGATAAGCGGCGC | GATCACCGTTCCCTCCGCCTGC | 25.1 |
| 31 | RM274 | 5 | CCTCGCTTATGAGAGCTTCG | CTTCTCCATCACTCCCATGG | 26.8 |
| 32 | RM30 | 6 | GGTTAGGCATCGTCACGG | TCACCTCACCACACGACACG | 5.27 |
| 33 | RM19638 | 6 | CCACACTGTACCGGTCGAAGACG | CTCTACAACTTGCAGCCCTGTCAGC | 6.3 |
| 34 | RM19723 | 6 | TCATGAGTGCCCTACCTCAATCC | ACAATGTGGTGGTTCTCTGTTGC | 8 |
| 35 | RM287 | 6 | GGCTACACCTACACGCGAGAACC | AGATGCATGGAATGCCTGTTTGG | 16.7 |
| 36 | RM20544 | 6 | AGGATGGGCTTACGATTGACG | CTTTACACTACCAGCGTGTTAGAAGG | 26.9 |
| 37 | RM5711 | 7 | GGACGGAAGGAATACGTCTGTAGG | CTGTCCATGCATCCATCTCTAGC | 3.1 |
| 38 | RM21137 | 7 | GGGAGGAAGTCTATTTGTCTCTCG | ATCTGACGGCAAGGATAGTGG | 5 |
| 39 | RM21165 | 7 | AGTTGCTACTGGCAGTTTGTTTGG | CAAGGGTATGTGACCCTTATTGAGC | 5.5 |
| 40 | RM21171 | 7 | AGGACCGTCCATCAGATCGTACAGC | CGTGCGTTTCAACTGGTGATGG | 5.6 |
| 41 | RM1253 | 7 | CTGAACTTGCCTGAGAACTC | GACGACCTCTCCATGCTCG | 6.9 |
| 42 | RM21260 | 7 | CTGCACAACCAGGAGAAATTAAGC | CTGACCACTCTAGCTTGCCTACC | 7.3 |
| 43 | RM547 | 8 | TAGGTTGGCAGACCTTTTCG | GTCAAGATCATCCTCGTAGCG | 5.59 |
| 44 | RM1384 | 8 | TGGTACGGGAGAACTGGTACGC | AATCGAGCCAGCCTAGCAAGC | 11.84 |
| 45 | RM483 | 8 | CTTCCACCATAAAACCGGAG | ACACCGGTGATCTTGTAGCC | 11.92 |
| 46 | RM44 | 8 | ACGGGCAATCCGAACAACC | TCGGGAAAACCTACCCTACC | 13.13 |
| 47 | RM23819 | 9 | CAATTCATAACGTCGGTCCTTCC | ATGGCTCTGTTTGCTGCATGG | 5 |
| 48 | RM105 | 9 | GTCGTCGACCCATCGGAGCCAC | TGGTCGAGGTGGGGATCGGGTC | 12.55 |
| 49 | RM288 | 9 | CCGGTCAGTTCAAGCTCTG | ACGTACGGACGTGACGAC | 18.56 |
| 50 | RM242 | 9 | GGCCAACGTGTGTATGTCTC | TATATGCCAAGACGGATGGG | 18.81 |
| 51 | RM6404 | 10 | AGCCGAGAGAGGAGTAGAACAGG | TGCTCTACCTAAATACTGGGCCTACC | 2.4 |
| 52 | RM216 | 10 | GCATGGCCGATGGTAAAG | TGTATAAAACCACACGGCCA | 5.3 |
| 53 | RM184 | 10 | ATCCCATTCGCCAAAACCGGCC | TGACACTTGGAGAGCGGTGTGG | 16.36 |
| 54 | RM3510 | 10 | ATAGAATGCCATGGAGACCAACC | AGACGACGAAGGCAGATAAGTCG | 18.6 |
| 55 | RM4771 | 10 | ACGTTGATTTCATTCAGGTC | ACGCTAACTGAGAAACATGG | 23.01 |
| 56 | RM26063 | 11 | GATCCATATGCCTCTTCGATTGG | AACTCCAGCAGTGAGAGCGTAGC | 2.2 |
| 57 | RM4469 | 11 | CCTATGCGTAGGTTCTCATTGTGC | AAGGGAGGGACGTAGAATAGATTTGG | 6.1 |
| 58 | RM202 | 11 | TGGAACACCCATAGACAAACAGC | TGGCAAGTGGTATTCTTCCTTCC | 8.9 |
| 59 | RM224 | 11 | ATCGATCGATCTTCACGAGG | TGCTATAAAAGGCATTCGGG | 21.8 |
| 60 | RM27096 | 11 | AGTTAGGATCGCTTCCAGGTTCC | TCCAACTGGAATATCGTCTTGTAGGC | 23.4 |
| 61 | RM27369 | 11 | ACATATCGACGGTGGATGAGAGC | TCCGTGTGCATACATTCTTGAGC | 28.1 |
| 62 | RM27466 | 12 | CAACAACAACGCGGTTTATTCC | GGCGTAATATTCCAAGAAGAGACG | 1.1 |
| 63 | RM2935 | 12 | CTTTGCACATGCGTCGTTGC | AAACTGCACATGGCCTCTCAGG | 7.4 |
| 64 | RM28424 | 12 | TCCACACACTTCGCCAATAAACC | CCGCCACCACTCCTCTATCC | 22.4 |
| 65 | RM28511 | 12 | AAAGAAAGCACCATGGCCACACC | CAGCATCTCCATCTCCATGAGTCC | 23.5 |
| 66 | RM1227 | 12 | CATGGTAGCACACACCCTTG | CATCGACATGTGGACCACTC | 27.3 |
