# Supplementary material for: DRR Dhan 58, a Seedling Stage Salinity Tolerant NIL of Improved Samba Mahsuri Shows Superior Performance in Multi-location Trials
Source: Rice (N Y). 2022 Aug 17;15:45. doi: 10.1186/s12284-022-00591-3 (PMC9385912; doi:10.1186/s12284-022-00591-3)
Supplement: Supplementary file 3 — Additional file 3. Table S2A: Estimation of Na and K in the introgressed lines of ISM with respect to recurrent and donor parents (stressed). Table S2B: Estimation of Na and K in the introgressed lines of ISM with respect to recurrent and donor parents (unstressed). [file 12284_2022_591_MOESM3_ESM.docx]

**Additional file 3: Table S2A:** Estimation of Na and K in the introgressed lines of ISM with respect to recurrent and donor parents (Stressed)

| **S. No** | **Designation** | **Shoot** | | | | | | | **Root** | | | | | |
| --- | --- | --- | --- | --- | --- | --- | --- | --- | --- | --- | --- | --- | --- | --- |
|  |  | TSL | TSPAD | TSFW | TSDW | TSNa | TSK | TSNa/K | TRL | TRFW | TRDW | TRNa | TRK | TRNa/K |
| 1 | ISM | 28.6 ± 0.2 | 14.9 ± 0.2 | 0.12 ± 0.1 | 0.04 ± 0.2 | 20.3 ± 0.1 | 56.7 ± 0.9 | 0.64 ± 0.4 | 4.5 ± 0.2 | 0.12 ± 0.0 | 0.04 ± 0.1 | 18.1 ± 0.4 | 46.2 ± 0.2 | 0.06 ± 0.1 |
| 2 | FL478 | 47.3 ± 0.3 | 26.4 ± 0.3 | 0.19 ± 0.2 | 0.11± 0.1 | 19.7 ± 0.1 | 90.8 ± 0.6 | 0.31 ± 0.2 | 13.5 ± 0.3 | 0.19 ± 0.0 | 0.11± 0.2 | 12.9 ± 0.2 | 67.5 ± 0.2 | 0.04 ± 0.2 |
| 3 | RP6287-12 | 30.5 ± 0.2 | 26.6 ± 0.2 | 0.1 ± 0.3 | 0.10 ± 0.2 | 19.3 ± 0.1 | 85.7 ± 0.3 | 0.28 ± 0.2 | 8.3 ± 0.1 | 0.1 ± 0.0 | 0.10 ± 0.2 | 12.7 ± 0.1 | 67.4 ± 0.1 | 0.04 ± 0.2 |
| 4 | RP6287-43 | 32.0 ± 0.3 | 25.3 ± 0.1 | 0.1 ± 0.2 | 0.10± 0.2 | 19.0 ± 0.0 | 85.2 ± 0.3 | 0.27 ± 0.2 | 7.6 ± 0.1 | 0.1 ± 0.0 | 0.10± 0.2 | 12.7 ± 0.0 | 67.4 ± 0.1 | 0.04 ± 0.2 |
| 5 | RP6287-88 | 44.0 ± 0.1 | 22.0 ± 0.1 | 0.1 ± 0.1 | 0.10 ± 0.2 | 19.4 ± 0.2 | 87.4 ± 0.8 | 0.28 ± 0.2 | 10.2 ± 0.1 | 0.1 ± 0.0 | 0.10 ± 0.3 | 12.4 ± 0.0 | 67.5 ± 0.0 | 0.04 ± 0.1 |
| 6 | RP6287-178 | 43.5 ± 0.2 | 20.6 ± 0.2 | 0.2 ± 0.4 | 0.10± 0.1 | 19.4 ± 0.0 | 85.0 ± 0.7 | 0.25 ± 0.1 | 11.3 ± 0.1 | 0.2 ± 0.0 | 0.10 ± 0.1 | 12.7 ± 0.1 | 67.2 ± 0.1 | 0.04 ± 0.2 |
| **CD%** | | 0.86 | 0.82 | 0.031 | 0.030 | 0.4 | 2.57 | 0.05 | 0.69 | 0.027 | 0.020 | 0.12 | 0.51 | 0.008 |
| **CV%** | | 0.96 | 1.51 | 1.39 | 4.89 | 0.83 | 1.24 | 6.66 | 3.13 | 7.68 | 12.18 | 2.45 | 0.31 | 8.04 |
| **PCV** | | 24.71 | 17.86 | 14.76 | 8.65 | 2.17 | 14.07 | 40.21 | 34.39 | 16.29 | 32.66 | 7.78 | 0.76 | 18.49 |
| **GCV** | | 24.69 | 17.80 | 14.69 | 7.13 | 2.00 | 14.01 | 39.66 | 34.25 | 14.37 | 30.30 | 6.55 | 0.69 | 16.65 |
| ***F*** | | 1953.64 | 415.30 | 333.70 | 7.37 | 18.10 | 378.89 | 107.14 | 358.95 | 11.49 | 19.54 | 13.56 | 16.52 | 13.85 |
| ***P value* (<0.05)** | | **<0.0001** | **<0.0001** | **<0.0001** | **<0.0017** | **<0.0001** | **<0.0001** | **<0.0001** | **<0.0001** | **<0.0002** | **<0.0001** | **<0.0001** | **<0.0001** | **<0.0001** |

TSL-Treated shoot length; TSPAD-Treated SPAD; TSFW- Treated fresh shoot weight; TSDW- Treated shoot dry weight; TSN-Treated shoot sodium; TSK- Treated shoot potassium; TSN/K- Treated shoot Na^+^/K^+^ ratio; TRL-Treated root length; TRFW- Treated fresh root weight; TRDW- Treated root dry weight; TRN-Treated root sodium; TSK- Treated root potassium; TRN/K- Control root Na^+^/K^+^ ratio

**Additional File 3: Table S2B:** Estimation of Na and K in the introgressed lines of ISM with respect to recurrent and donor parents (Unstressed)

| **S. No** | **Designation** | **Shoot** | | | | | | | **Root** | | | | | |
| --- | --- | --- | --- | --- | --- | --- | --- | --- | --- | --- | --- | --- | --- | --- |
|  |  | CSL | CSPAD | CSFW | CSDW | CSNa | CSK | CSNa/K | CRL | CRFW | CRDW | CRNa | CRK | CRNa/K |
| 1 | ISM | 38.6 ± 0.2 | 17.5 ± 0.2 | 3.0 ± 0.0 | 0.3 ± 0.0 | 36.5 ± 0.9 | 50.2 ± 0.3 | 0.29 ± 0.0 | 10.1 ± 0.2 | 0.9 ± 0.0 | 0.1 ± 0.0 | 26.7 ± 0.3 | 21.4 ± 0.3 | 1.19 ± 0.1 |
| 2 | FL478 | 47.3 ± 0.3 | 34.5 ± 0.3 | 2.8 ± 0.0 | 0.6 ± 0.0 | 28.7 ± 0.8 | 47.3 ± 0.3 | 0.42 ± 0.0 | 15.3 ± 0.2 | 1.0 ± 0.0 | 0.2 ± 0.0 | 20.3 ± 0.2 | 29.2 ± 0.2 | 0.71 ± 0.2 |
| 3 | RP6287-12 | 40.5 ± 0.1 | 26.6 ± 0.2 | 3.0 ± 0.0 | 0.5 ± 0.0 | 23.6 ± 1.0 | 48.6 ± 0.7 | 0.4 ± 0.0 | 11.3 ± 0.1 | 0.9 ± 0.0 | 0.1 ± 0.0 | 22.0 ± 0.0 | 27.0 ± 0.1 | 0.9 ± 0.2 |
| 4 | RP6287-43 | 44.0 ± 0.1 | 28.6 ± 0.2 | 3.0 ± 0.0 | 0.5 ± 0.0 | 23.2 ± 0.9 | 48.8 ± 0.4 | 0.4 ± 0.0 | 11.7 ± 0.1 | 0.9 ± 0.0 | 0.1 ± 0.0 | 22.1 ± 0.3 | 26.2 ± 0.2 | 0.9 ± 0.1 |
| 5 | RP6287-88 | 45.6 ± 0.1 | 33.6 ± 0.2 | 1.8 ± 0.0 | 0.5 ± 0.0 | 24.9 ± 0.5 | 46.6 ± 1.0 | 0.4 ± 0.0 | 13.2 ± 0.1 | 0.9 ± 0.0 | 0.1 ± 0.0 | 21.7 ± 0.1 | 27.2 ± 0.1 | 0.8 ± 0.2 |
| 6 | RP6287-178 | 46.3 ± 0.1 | 33.1 ± 0.1 | 3.0 ± 0.0 | 0.5 ± 0.0 | 21.5 ± 0.4 | 47.0 ± 0.0 | 0.4 ± 0.0 | 13.3 ± 0.2 | 0.9± 0.0 | 0.1 ± 0.0 | 22.2 ± 0.1 | 27.4 ± 0.6 | 0.8 ± 0.2 |
| **CD%** | | 0.76 | 0.63 | 0.04 | 0.03 | 3.76 | 2.9 | 0.02 | 0.65 | 0.07 | 0.01 | 0.33 | 1.05 | 0.024 |
| **CV%** | | 0.75 | 0.91 | 0.94 | 2.82 | 5.63 | 2.25 | 2.14 | 2.16 | 4.16 | 5.37 | 0.58 | 1.59 | 1.14 |
| **PCV** | | 18.08 | 23.66 | 25.56 | 23.09 | 19.76 | 16.59 | 12.30 | 16.68 | 17.45 | 3.24 | 9.07 | 9.28 | 17.04 |
| **GCV** | | 18.06 | 23.65 | 25.54 | 22.92 | 18.94 | 16.44 | 12.11 | 16.54 | 14.30 | 2.41 | 9.05 | 9.15 | 17.00 |
| ***F*** | | 1738.73 | 2025.34 | 2182.99 | 198.42 | 34.96 | 161.27 | 97.14 | 176.99 | 233.33 | 3.6 | 719.91 | 99.31 | 668.38 |
| ***P value* (<0.05)** | | **<0.0001** | **<0.0001** | **<0.0001** | **<0.0001** | **<0.0001** | **<0.0001** | **<0.0001** | **<0.0001** | **<0.0001** | **<0.0281** | **<0.0001** | **<0.0001** | **<0.0001** |

CSL-Control shoot length; CSPAD-Control SPAD (Soil Plant Analysis Development chlorophyll meter); CSFW- Control fresh shoot weight; CSDW- Control shoot dry weight; CSN-Control shoot sodium; CSK- Control shoot potassium; CSN/K Control shoot Na^+^/K^+^ ratio; CRL-Control root CRFW- Control fresh root weight; CRDW- Control root dry weight; CRN-Control root sodium; CSK- Control root potassium; CRN/K- Control root Na^+^/K^+^ ratio.
