# Supplementary material for: DRR Dhan 58, a Seedling Stage Salinity Tolerant NIL of Improved Samba Mahsuri Shows Superior Performance in Multi-location Trials
Source: Rice (N Y). 2022 Aug 17;15:45. doi: 10.1186/s12284-022-00591-3 (PMC9385912; doi:10.1186/s12284-022-00591-3)
Supplement: Supplementary file 4 — Additional file 4. Table S3: Station trial data of promising entries during wet season 2018 (June to Nov). [file 12284_2022_591_MOESM4_ESM.docx]

**Additional File 4: Table S3:** Station trial data of promising entries during wet season 2018 (June to November)

| **Entry** | **Yield/ha** | | | **Mean yield (Kg/ha)** |
| --- | --- | --- | --- | --- |
|  | **Rep 1** | **Rep 2** | **Rep 3** |  |
| ISM | 4712 | 4814 | 4614 | 4713 |
| RP6287-12 | 5123 | 5214 | 5066 | 5134 |
| RP6287-43 | 4545 | 4487 | 4665 | 4567 |
| DRR Dhan58 | 5720 | 5810 | 5780 | 5770 |
| RP6287-178 | 4989 | 4686 | 4876 | 4850 |

The station trial data was conducted at ICAR-IIRR, Hyderabad during wet season 2018 and the data is a mean data of three replications
