# Supplementary material for: DRR Dhan 58, a Seedling Stage Salinity Tolerant NIL of Improved Samba Mahsuri Shows Superior Performance in Multi-location Trials
Source: Rice (N Y). 2022 Aug 17;15:45. doi: 10.1186/s12284-022-00591-3 (PMC9385912; doi:10.1186/s12284-022-00591-3)
Supplement: Supplementary file 5 — Additional file 5. Table S4: Site characterization of tested location data during Kharif 2019 and 2020 [file 12284_2022_591_MOESM5_ESM.docx]

**Additional File 6: Table S5**: Summary of yield (kg/ha) data of Zone wise Advanced varietal trails (AVT NIL)

for Kharif 2019 and 2020 (June to November)

| Mean yield (kg/ha) | Season of **testing** | **Yield under normal conditions** | | | | **Coastal salinity yield trials**  **(under stress prone conditions)** | | | |
| --- | --- | --- | --- | --- | --- | --- | --- | --- | --- |
|  |  | No of trials | DRR Dhan58 | ISM (RP) | **% Increase over RP** | **No of trials** | **DRR Dhan58** | **ISM (RP)** | **% Increase over RP** |
| Odisha (Zone III) | Kharif 2019  (AVT-1) | 2 | 5751 | 4861 | + 18.31 | 1 | 3141 | 2707 | + 16.03 |
|  | Kharif 2020 (AVT-2) | 3 | 4079 | 3291 | + 23.94 | - | - | - | - |
| Odisha wt. avg. | | **5** | 4748 | 3919 | + 21.15 | **1** | 3141 | 2707 | **+ 16.03** |
| Bihar (Zone III) | Kharif 2019 (AVT-1) | - | - | - | - | - | - | - | **-** |
|  | Kharif 2020(AVT-2) | 1 | 5026 | 3545 | + 41.78 | - | - | - | **-** |
| Bihar wt. avg. | | **1** | 5026 | 3545 | + 41.78 | - | - | - | **-** |
| Chhattisgarh (Zone V) | Kharif 2019 (AVT-1) | 1 | 3510 | 3093 | + 13.49 | - | - | - | **-** |
|  | Kharif 2020(AVT-2) | 2 | 3066 | 3503 | - 12.48 | - | - | - | **-** |
| Chhattisgarh wt. avg. | | **3** | 3214 | 3316 | - 4.53 | - | - | - | - |
| Maharashtra (Zone V/VI) | Kharif 2019 (AVT-1) | 1 | 3902 | 3252 | + 19.99 | 1 | 1861 | 448 | + 315.40 |
|  | Kharif 2020(AVT-2) | 2 | 4603 | 4686 | - 1.77 | - | - | - | **-** |
| Maharashtra wt. avg. | | **3** | 4369 | 4208 | + 3.83 | **1** | 1861 | 448 | **+ 315.40** |
| Gujarat (Zone VI) | Kharif 2019 (AVT-1) | 1 | 5046 | 4431 | + 13.88 | - | - | - | **-** |
|  | Kharif 2020(AVT-2) | 1 | 4478 | 4783 | - 6.38 | - | - | - | **-** |
| Gujarat wt. avg. | | **2** | 4762 | 4601 | + 3.4 | **-** | - | - | - |
| Andhra Pradesh(Zone VII) | Kharif 2019 (AVT-1) | - | - | - | - | 1 | 5431 | 4680 | + 16.05 |
|  | Kharif 2020(AVT-2) | 4 | 3586 | 3629 | - 1.18 | 1 | 2234 | 216 | + 934.26 |
| Andhra Pradesh wt. avg. | | **4** | 3586 | 3629 | - 1.18 | **2** | 3832 | 2448 | **+ 56.54** |
| Telangana (Zone VII) | Kharif 2019 (AVT-1) | - | - | - | - | - | - | - | **-** |
|  | Kharif 2020(AVT-2) | 1 | 3265 | 3175 | + 2.83 | - | - | - | **-** |
| Telangana wt. avg. | | **1** | 3265 | 3175 | + 2.83 | **-** | **-** | - | - |
