# Supplementary material for: DRR Dhan 58, a Seedling Stage Salinity Tolerant NIL of Improved Samba Mahsuri Shows Superior Performance in Multi-location Trials
Source: Rice (N Y). 2022 Aug 17;15:45. doi: 10.1186/s12284-022-00591-3 (PMC9385912; doi:10.1186/s12284-022-00591-3)
Supplement: Supplementary file 6 — Additional file 6. Table S6: Sequencing and mapping statistics of the rice lines used in this study with respect to the Nipponbare reference genome [file 12284_2022_591_MOESM6_ESM.docx]

| **Sample** | **Source** | **Total reads** | **Mapped reads** | **% Mapped reads** | **% Reference coverage** | **Sequencing depth (X)** |
| --- | --- | --- | --- | --- | --- | --- |
| Pokkali | This study | 171,267,514 | 150,861,483 | 88.09 | 93.13 | 57.4 |
| ISM | This study | 184,584,204 | 165,609,846 | 89.72 | 93.57 | 63.1 |
| DRR Dhan58 | This study | 228,285,350 | 201,163,969 | 88.12 | 93.91 | 76.4 |
| FL478 | SRR9943932 | 52,402,002 | 50,866,566 | 97.06 | 91.84 | 13.4 |

**Additional file 7: Table S6:** Sequencing and mapping statistics of the rice lines used in this study with respect to the Nipponbare reference genome
