# Supplementary material for: DRR Dhan 58, a Seedling Stage Salinity Tolerant NIL of Improved Samba Mahsuri Shows Superior Performance in Multi-location Trials
Source: Rice (N Y). 2022 Aug 17;15:45. doi: 10.1186/s12284-022-00591-3 (PMC9385912; doi:10.1186/s12284-022-00591-3)
Supplement: Supplementary file 7 — Additional file 7. Table S7: Distribution of variants (SNPs, Insertions and Deletions) across the chromosomes in the rice lines including Pokkali, FL478, ISM, and DRR Dhan 58. [file 12284_2022_591_MOESM7_ESM.docx]

**Additional file 8: Table S7**: Distribution of variants (SNPs, Insertions and Deletions) across the chromosomes in the rice lines including Pokkali, FL478, ISM, and DRR Dhan 58.

| **Chromo**  **some** | **Pokkali** | | | **FL478** | | | **ISM** | | | **DRR Dhan58** | | |
| --- | --- | --- | --- | --- | --- | --- | --- | --- | --- | --- | --- | --- |
|  | **SNPs** | **Insertions** | **Deletions** | **SNPs** | **Insertions** | **Deletions** | **SNPs** | **Insertions** | **Deletions** | **SNPs** | **Insertions** | **Deletions** |
| **Chr1** | 81,833 | 13,023 | 12,722 | 82,584 | 11,818 | 12,258 | 86,938 | 13,581 | 13,222 | 94,599 | 14,272 | 14,056 |
| **Chr2** | 74,192 | 11,130 | 11,027 | 72,338 | 9,867 | 10,202 | 75,509 | 11,328 | 11,155 | 82,589 | 11,924 | 11,709 |
| **Chr3** | 71,767 | 11,093 | 10,974 | 69,151 | 9,622 | 10,003 | 76,988 | 11,899 | 11,603 | 83,311 | 12,441 | 12,047 |
| **Chr4** | 53,039 | 7,610 | 7,701 | 50,529 | 6,502 | 6,801 | 55,691 | 7,750 | 7,723 | 60,362 | 8,136 | 8,088 |
| **Chr5** | 59,916 | 8,609 | 8,491 | 53,795 | 7,135 | 7,110 | 55,614 | 8,231 | 8,020 | 59,569 | 8,531 | 8,413 |
| **Chr6** | 59,146 | 8,765 | 8,819 | 55,683 | 7,273 | 7,751 | 61,808 | 9,108 | 8,963 | 66,890 | 9,491 | 9,371 |
| **Chr7** | 53,729 | 7,881 | 7,975 | 51,384 | 6,657 | 7,146 | 53,278 | 7,732 | 7,741 | 60,319 | 8,367 | 8,364 |
| **Chr8** | 54,195 | 7,536 | 7,513 | 51,166 | 6,403 | 6,790 | 54,376 | 7,442 | 7,346 | 61,744 | 8,000 | 7,985 |
| **Chr9** | 43,593 | 6,290 | 6,069 | 41,670 | 5,398 | 5,556 | 46,627 | 6,551 | 6,368 | 51,302 | 6,956 | 6,721 |
| **Chr10** | 46,011 | 6,456 | 6,348 | 44,236 | 5,622 | 5,756 | 47,703 | 6,511 | 6,501 | 53,497 | 6,966 | 6,958 |
| **Chr11** | 50,705 | 7,205 | 7,098 | 50,292 | 6,493 | 6,701 | 53,086 | 7,349 | 7,389 | 57,801 | 7,710 | 7,781 |
| **Chr12** | 42,097 | 6,069 | 5,999 | 42,164 | 5,494 | 5,639 | 47,321 | 6,560 | 6,450 | 57,576 | 7,184 | 7,334 |
| **Total** | 6,90,223 | 1,01,667 | 1,00,736 | 6,64,992 | 88,284 | 91,713 | 7,14,939 | 1,04,042 | 1,02,481 | 7,89,559 | 1,09,978 | 1,08,827 |
