# Supplementary material for: DRR Dhan 58, a Seedling Stage Salinity Tolerant NIL of Improved Samba Mahsuri Shows Superior Performance in Multi-location Trials
Source: Rice (N Y). 2022 Aug 17;15:45. doi: 10.1186/s12284-022-00591-3 (PMC9385912; doi:10.1186/s12284-022-00591-3)
Supplement: Supplementary file 8 — Additional file 8: Table S8: Distribution of variants based on the location in the genome of the rice lines and their consequences. [file 12284_2022_591_MOESM8_ESM.docx]

**Additional file 9: Table S8**: Distribution of variants based on the location in the genome of the rice lines and their consequences

| **Variant Location/Consequences** | **Pokkali** | **FL478** | **ISM** | **DRR Dhan58** |
| --- | --- | --- | --- | --- |
| UPSTREAM | 339,904 | 321,371 | 363,702 | 398,747 |
| DOWNSTREAM | 331,772 | 315,164 | 345,377 | 377,012 |
| INTRON | 246,701 | 240,889 | 244,012 | 262,354 |
| INTERGENIC | 528,409 | 514,690 | 556,576 | 614,771 |
| UTR_3_PRIME | 44,075 | 40,947 | 43,234 | 45,815 |
| UTR_5_PRIME | 31,017 | 24,770 | 31,476 | 32,892 |
| EXON | 152,827 | 133,181 | 153,962 | 166,087 |
| SPLICE_SITE_ACCEPTOR | 524 | 429 | 546 | 590 |
| SPLICE_SITE_DONOR | 533 | 472 | 528 | 564 |
| SPLICE_SITE_REGION | 8,424 | 7,873 | 8,419 | 8,951 |
| MISSENSE | 79,264 | 70,503 | 79,847 | 86,624 |
| NONSENSE | 2,306 | 2,008 | 2,362 | 2,669 |
| SILENT | 59,900 | 52,355 | 60,250 | 64,601 |
| FRAMESHIFT | 5,076 | 4,282 | 5,181 | 5,605 |
