# Supplementary material for: DRR Dhan 58, a Seedling Stage Salinity Tolerant NIL of Improved Samba Mahsuri Shows Superior Performance in Multi-location Trials
Source: Rice (N Y). 2022 Aug 17;15:45. doi: 10.1186/s12284-022-00591-3 (PMC9385912; doi:10.1186/s12284-022-00591-3)
Supplement: Supplementary file 9 — Additional file 9. Table S5: Summary of yield (kg/ha) data of Zone wise Advanced varietal trails (AVT NIL) for Kharif 2019 and 2020 (June to Nov) [file 12284_2022_591_MOESM9_ESM.docx]

**Additional File 5: Table S4**: Site characterization of tested location data during Kharif 2019 and 2020

|  | **2019** | | **2020** | | **Condition**  **(Stress or Unstress)** | **Stress exposed to Seedling stage or reproductive stage** |
| --- | --- | --- | --- | --- | --- | --- |
| **Location/State** | **Soil pH** | **Soil EC (dSm^-1^)** | **Soil**  **pH** | **Soil EC**  **(dS/m)** |  |  |
| NRRI-Cuttack  (Odish) | 5.7 | 6.59 | 5.9 | 6.7 | Stress | It has been exposed to both seedling stage and reproductive stage. Hence Salinity affects almost all aspects of plant development including germination, Vegetative growth and reproductive development |
| Chinsurah  (West Bengal) | 6.5 | 3.5-4.5 | 6.5 | 3.5-4.5 | Stress |  |
| Canning  (West Bengal) | 6.7 | 5.43 | 6.7 | 6.43 | Stress |  |
| Machilipatnam  (Andhra Pradesh) | 9.17 | NA | 7.86 | 5.95 | Stress |  |
| Panvel  (Maharashtra) | 7-8 | 2.42, 2.49 & 2.57 | NA | NA | Stress |  |
| Brahmavar (Karnataka) | 5.5 | NA | 5.47 | 3.22 | Unstressed |  |
| Navsari  (Gujarat) | 8.2 | 5.8 | NA | NA | Unstressed |  |
| Karaikal | NA | NA | 7.75-  8.38 | 0.8-1.02 | Unstressed |  |
